# Supplementary material for: Gerrymandering in the heart: How tissue heterogeneities promote premature ventricular contractions
Source: Biophys J. 2026 Apr 6;125(9):2140–51. doi: 10.1016/j.bpj.2026.03.057 (PMC13351706; doi:10.1016/j.bpj.2026.03.057)
Supplement: Document S1. Figures S1–S8 [file mmc1.pdf]

Biophysical Journal, Volume *125*

Supplemental information

Gerrymandering in the heart: How tissue heterogeneities promote premature ventricular contractions

Daisuke Sato and Donald M. Bers

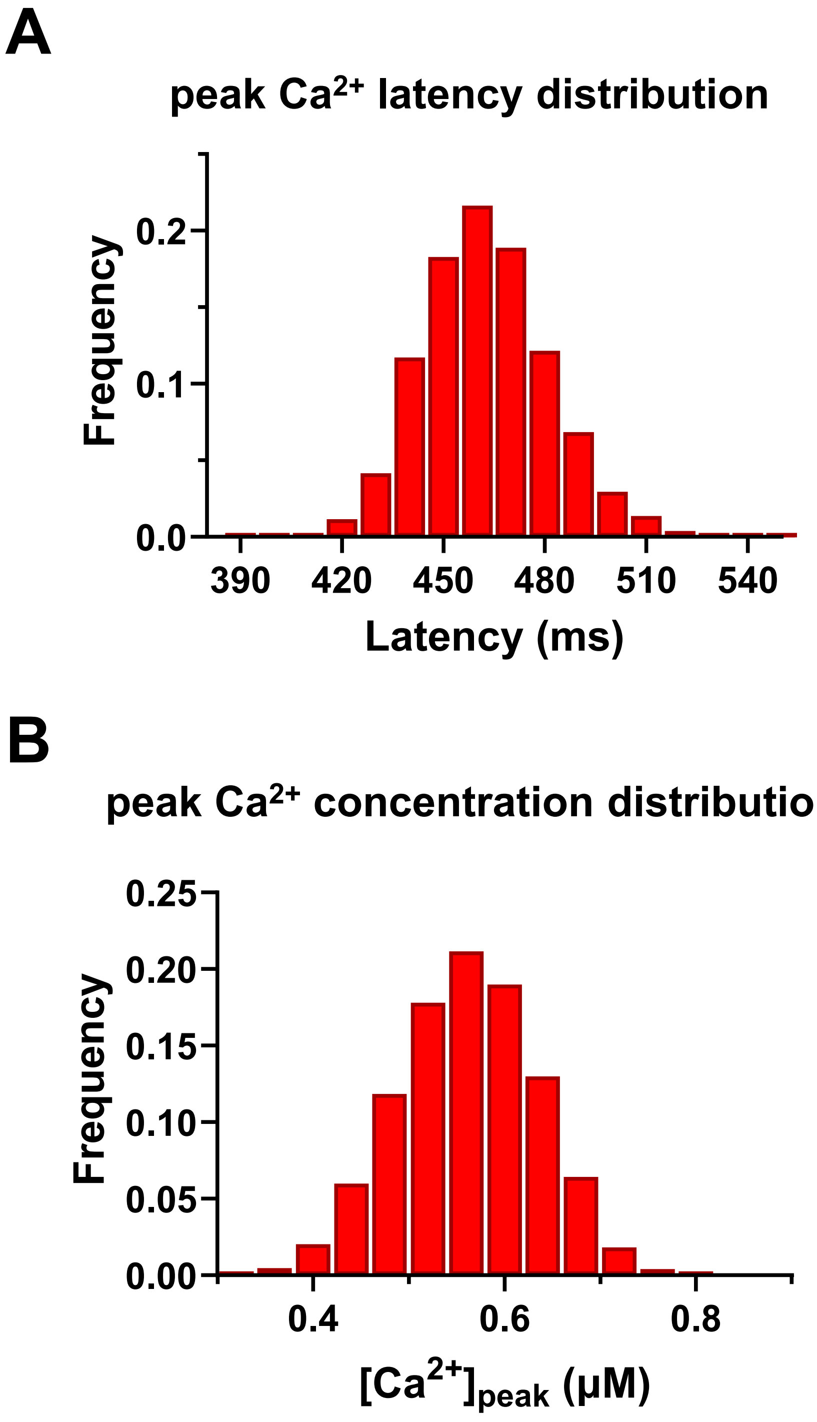

Figure S1. Characteristics of spontaneous Ca^{2+} waves. (A) The histogram of the latency of the peak $[Ca^{2+}]_i$ from the preceding pacing. The mean latency is 461.9 ms and the standard deviation is 17.5 ms. (B) The histogram of the peak Ca^{2+} transient amplitude. The mean peak Ca^{2+} transient is 0.56 μM and the standard deviation is 0.076 μM. These histograms were obtained from 300 simulations of DAD^+ cells.

Figure S1

Uncoupled tissue

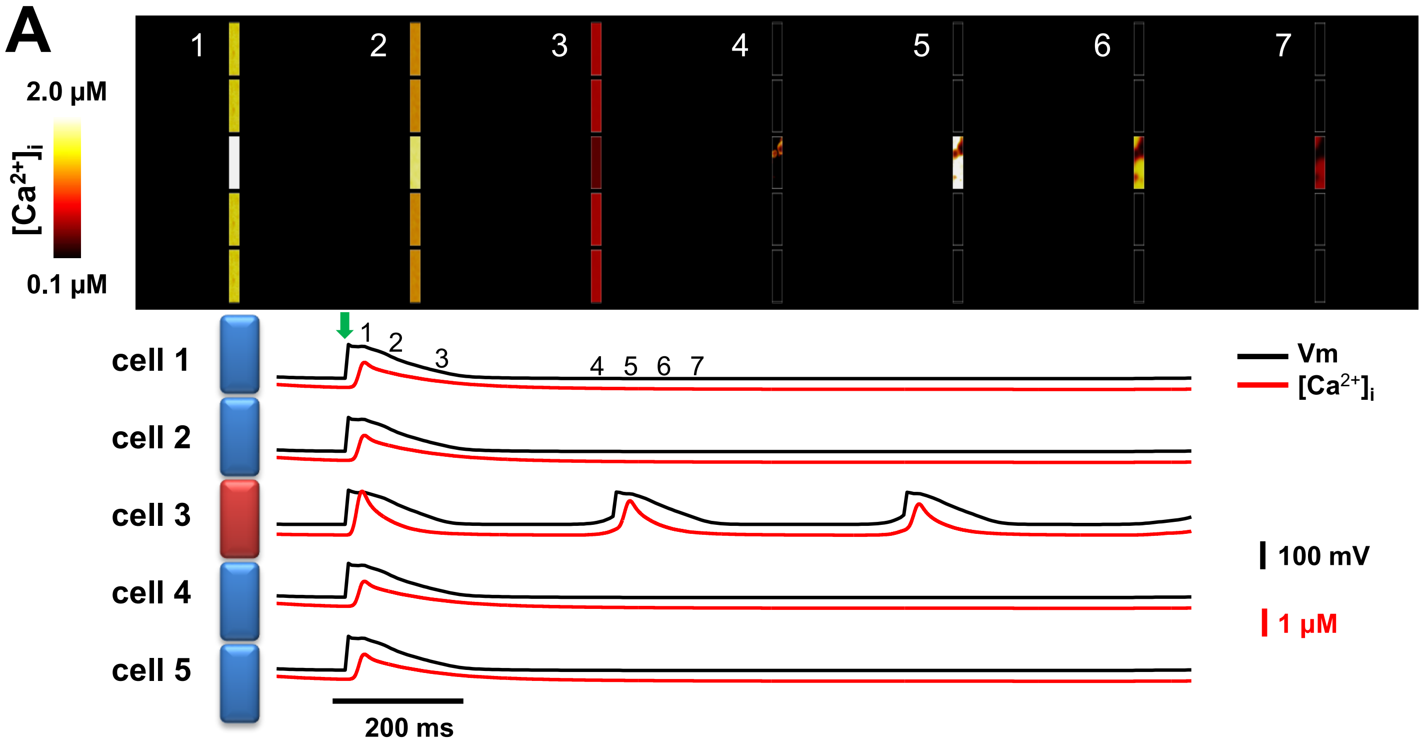

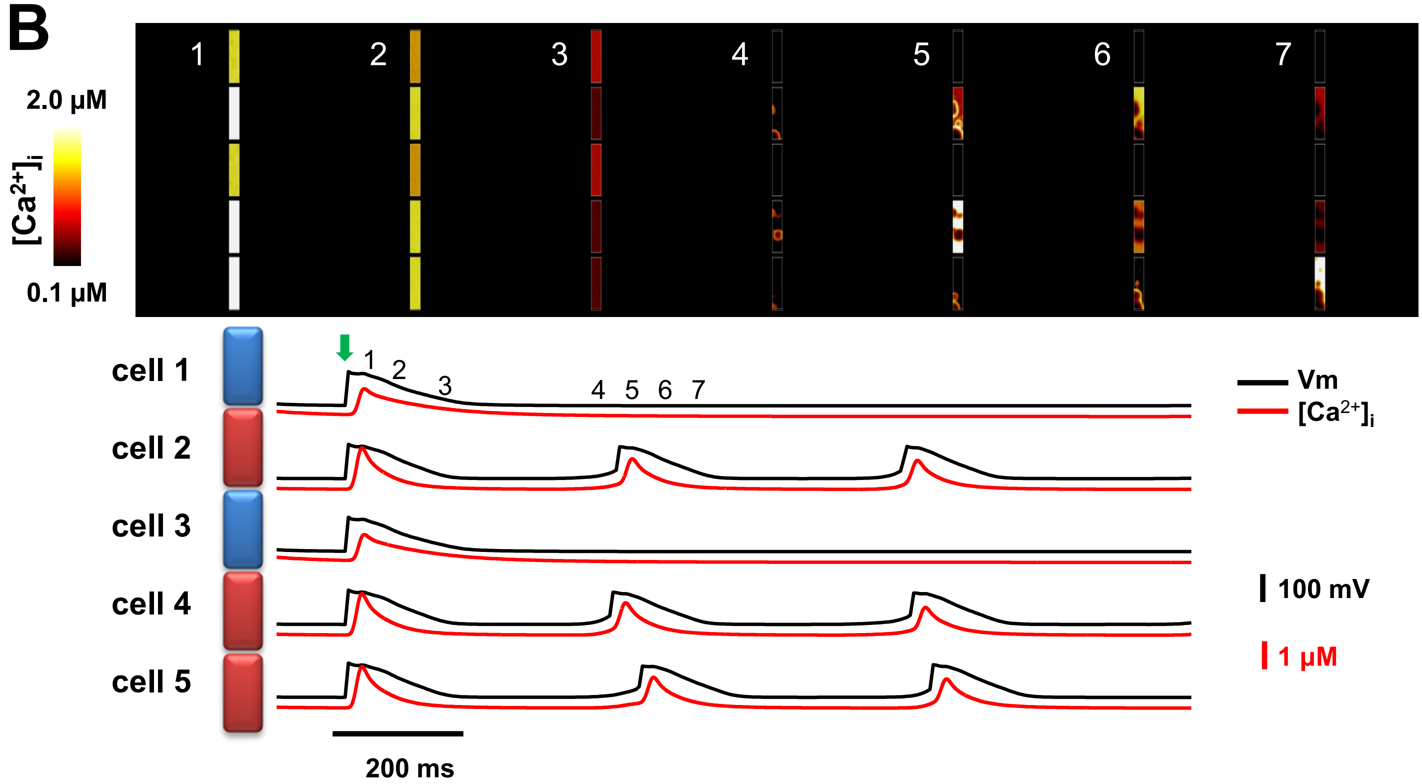

Figure S2. The uncoupled cases corresponding to Figures 1B and 1C. (A) The uncoupled case corresponding to Figure 1B. **(B)** The uncoupled case corresponding to Figure 1C.

Figure S2

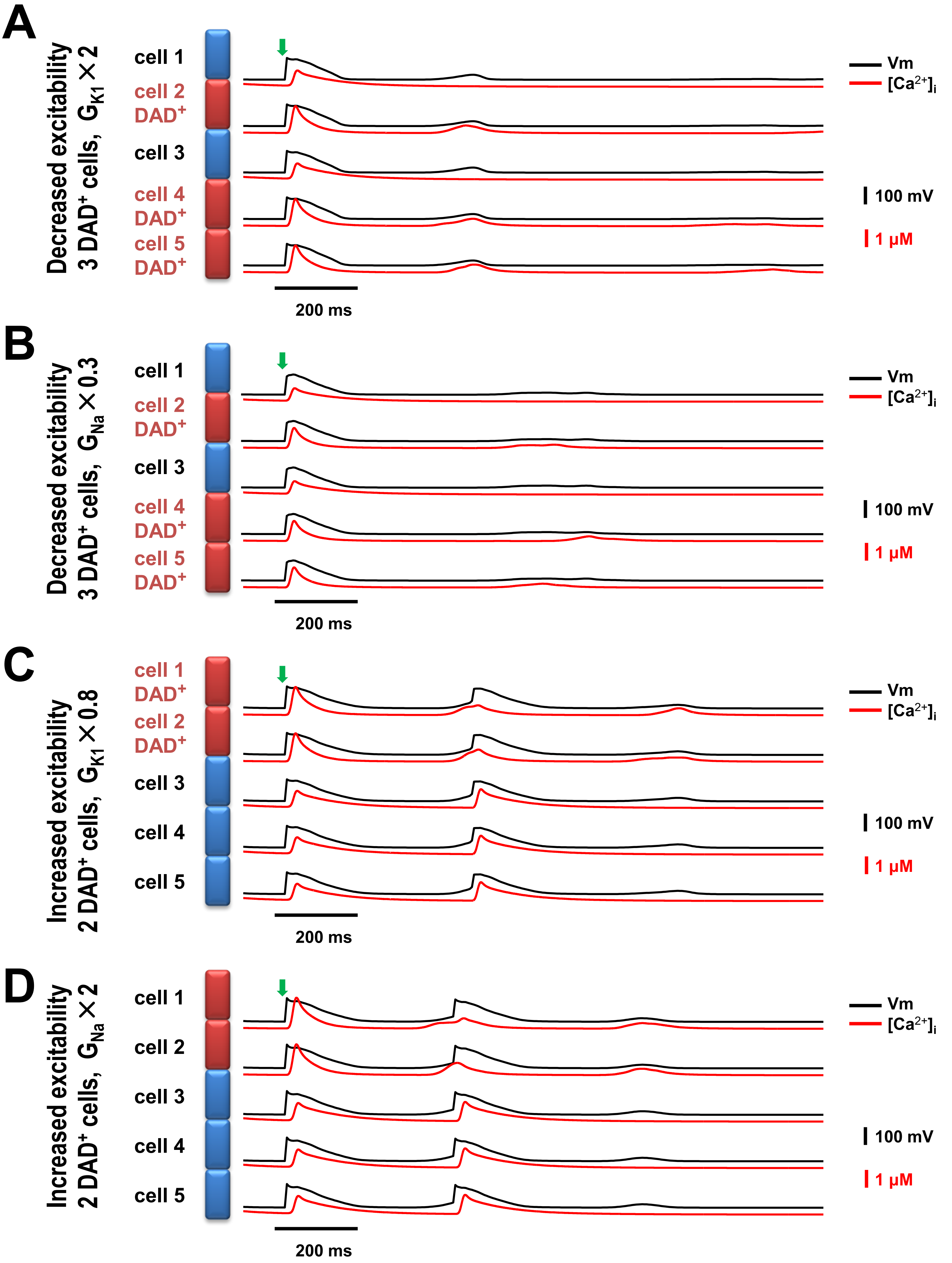
A
Decreased excitability
3 DAD⁺ cells, $G_{K1} \times 2$
cell 1
cell 2 DAD⁺
cell 3
cell 4 DAD⁺
cell 5 DAD⁺
Vm
$[Ca^{2+}]_i$
100 mV
1 μM
200 ms
B
Decreased excitability
3 DAD⁺ cells, $G_{Na} \times 0.3$
cell 1
cell 2 DAD⁺
cell 3
cell 4 DAD⁺
cell 5 DAD⁺
Vm
$[Ca^{2+}]_i$
100 mV
1 μM
200 ms
C
Increased excitability
2 DAD⁺ cells, $G_{K1} \times 0.8$
cell 1 DAD⁺
cell 2 DAD⁺
cell 3
cell 4
cell 5
Vm
$[Ca^{2+}]_i$
100 mV
1 μM
200 ms
D
Increased excitability
2 DAD⁺ cells, $G_{Na} \times 2$
cell 1
cell 2
cell 3
cell 4
cell 5
Vm
$[Ca^{2+}]_i$
100 mV
1 μM
200 ms

Figure S3

Figure S3. Cellular excitability modulates the critical mass required for a PVC. A reduction in cellular excitability increases the required number of DAD^+ cells for the initiation of a PVC. Conversely, an increase in cellular excitability reduces the required number of DAD^+ cells for a PVC. (A) Increasing I_{K1} current ($\times$ 2) to decrease excitability prevented PVCs, even with three DAD^+ cells. (B) Decreasing I_{Na} current ($\times$ 0.3) similarly prevented PVCs, even with three DAD^+ cells. (C) Decreasing I_{K1} current ($\times$ 0.3) to enhance excitability led to a PVC with only two DAD^+ cells. (D) An increase in I_{Na} ($\times$ 2) current also led to a PVC with two DAD^+ cells.

Figure S3

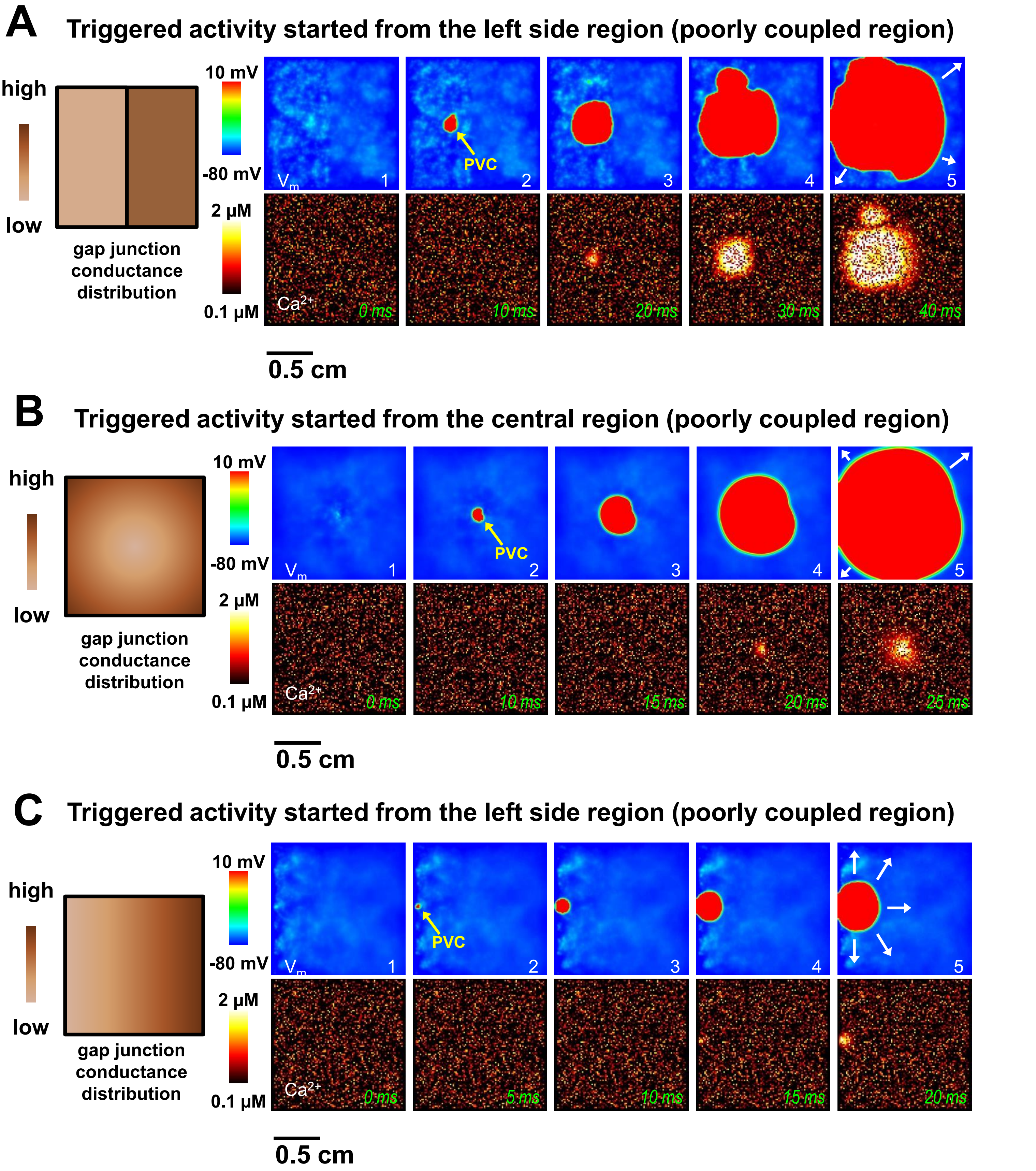

Figure S4. Additional simulations with different seeds of the random number generator for Figures 3B, 4A and 4B. (A) Simulation corresponding to Figure 3B. (see Supplementary Video S9) **(B)** Simulation corresponding to Figure 4A. (see Supplementary Video S10) **(C)** Simulation corresponding to Figure 4B. (see Supplementary Video S11)

Figure S4

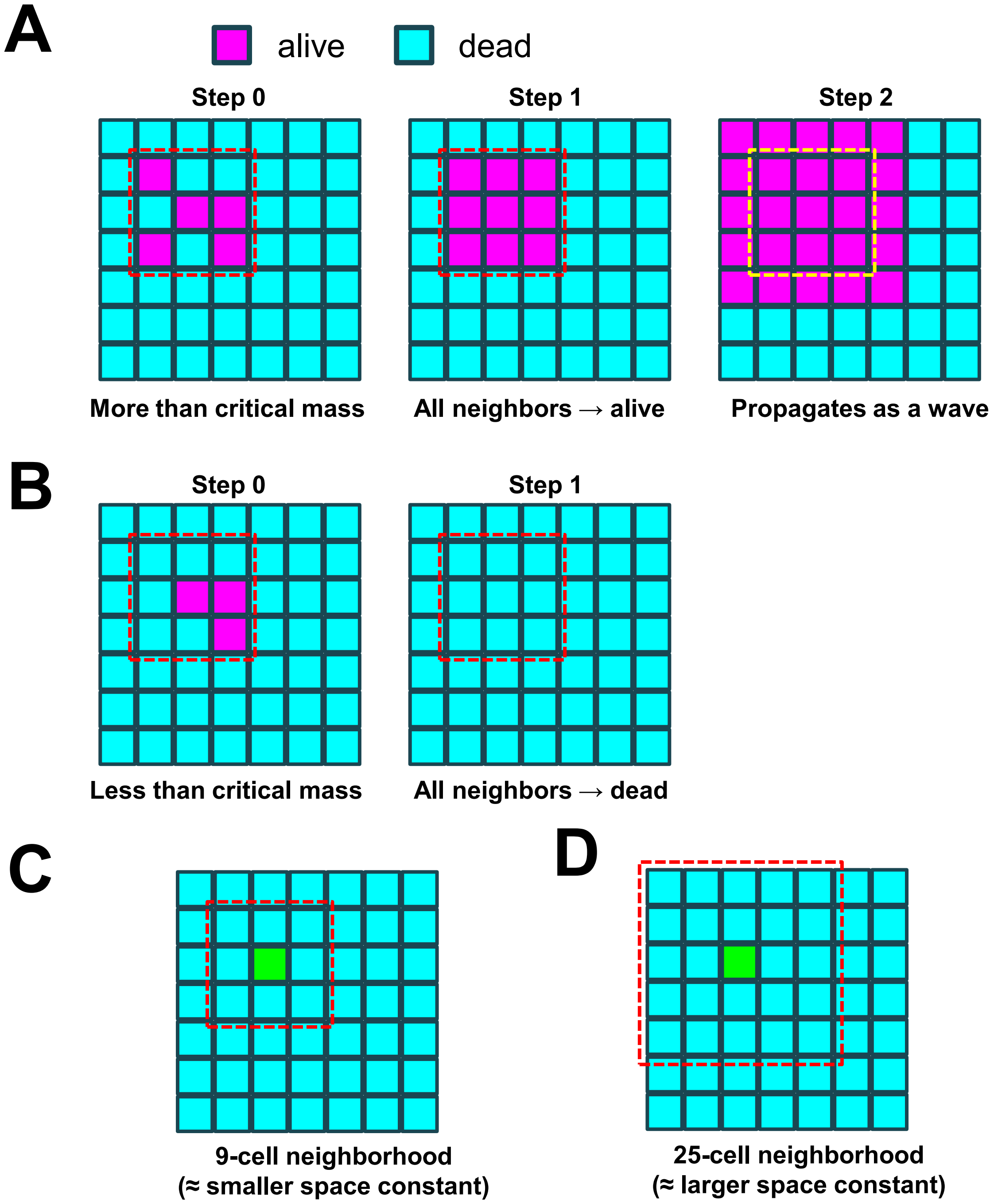

Figure S5. Cellular automaton model illustrating how the space constant influences PVC initiation. Magenta cells are "alive" and cyan cells are "dead." At each time step, a cell's state is determined by the percentage of "alive" cells in its local neighborhood (including itself). **(A)** If this percentage exceeds the 40% critical mass, all cells in the neighborhood become "alive." **(B)** Otherwise, they become "dead." **(C)** A 9-cell neighborhood (a cell plus its 8 immediate neighbors), representing a short space constant, meets the threshold if 4 or more cells are "alive" (4/9 ≈ 44%). A 25-cell neighborhood (a cell plus its 24 neighbors), representing a long space constant, meets the threshold if 10 or more cells are "alive" (10/25 = 40%).

Figure S5

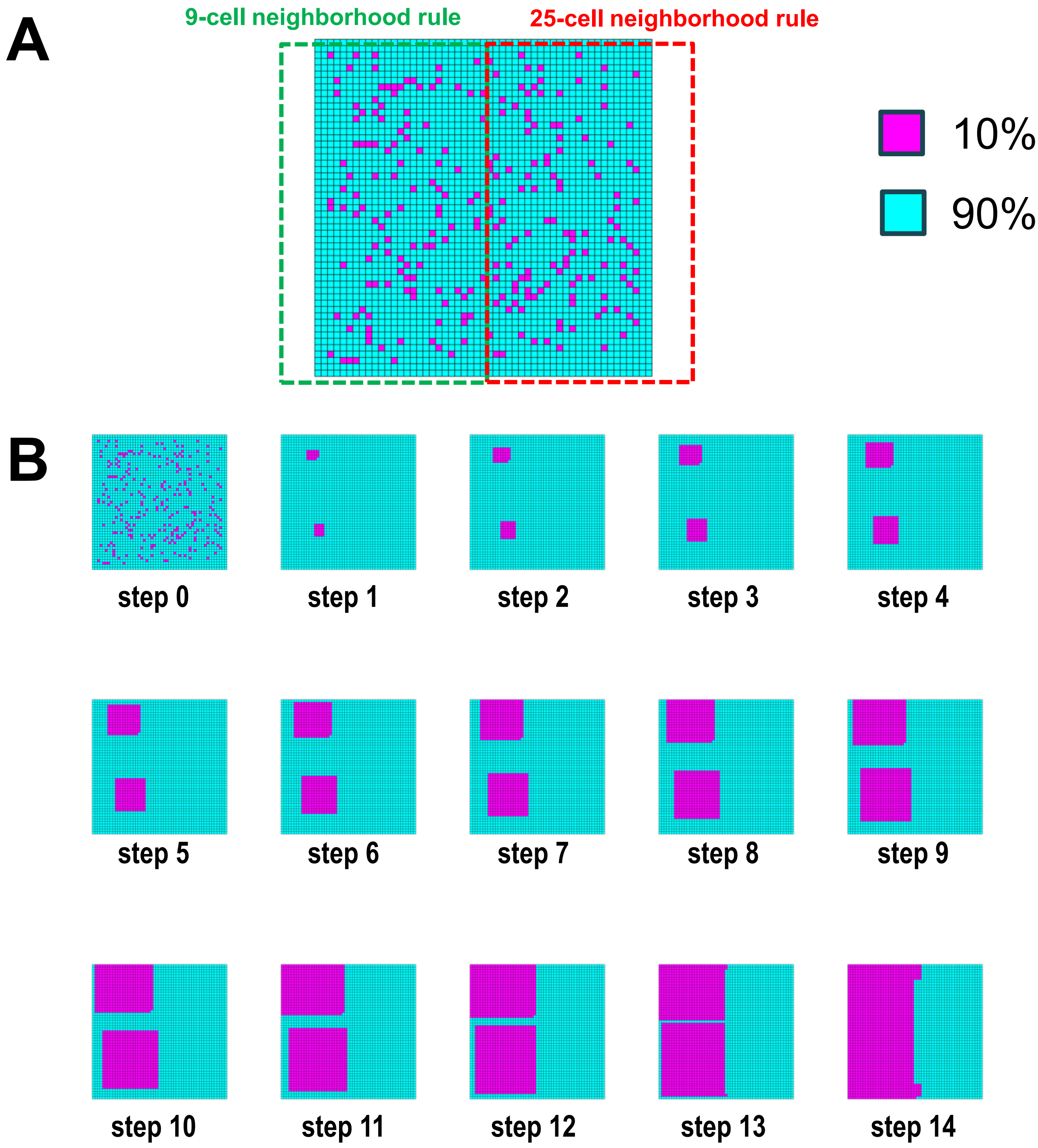

Figure S6. Simulation on a 50 × 50 grid with a heterogeneous neighborhood rule, based on the model in Figure S6. (A) The left half of the grid uses the 9-cell neighborhood rule, and the right half uses the 25-cell rule. This setup simulates tissue with a short space constant on the left and a long space constant on the right. **(B)** Images show time snapshots from a representative simulation starting with a random distribution of "alive" cells ("seeds"). The rule is applied iteratively, demonstrating how waves of "alive" cells can initiate and propagate across the grid.

Figure S6

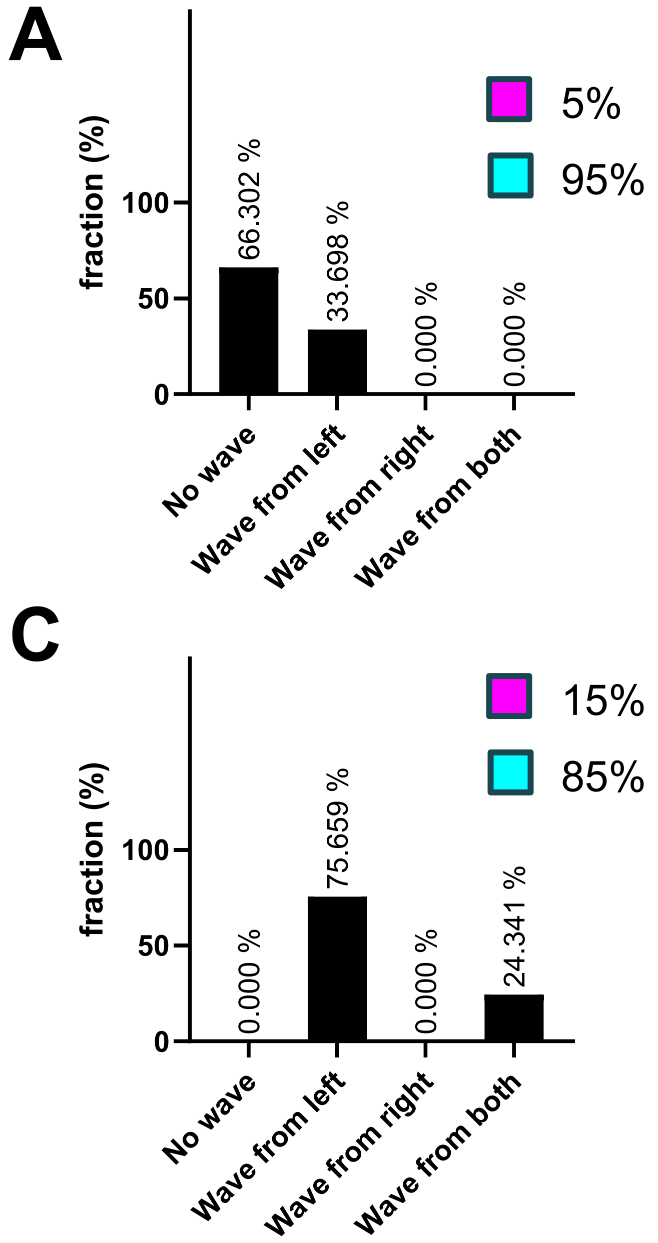

Figure S7. Summary of simulations described in Figure S6 with different random seeds, showing how the initial density of "alive" cells affects wave initiation. The percentage of initially "alive" cells is analogous to the density of pathological cells (DAD^+ cells) in tissue; a lower percentage represents healthier tissue. Simulations were run with initial "alive" cell densities ranging from 5% to 15%. **(A)** With 5% initially "alive," waves generally failed to form, and the grid tended toward a "dead" state. **(B)** With 10% initially "alive," waves were typically initiated on the left side (short space constant), because a smaller neighborhood increases the likelihood of randomly exceeding the critical mass threshold. **(C)** With 15% initially "alive," waves often initiated on the left side, but many also initiated simultaneously on the right side.

Figure S7

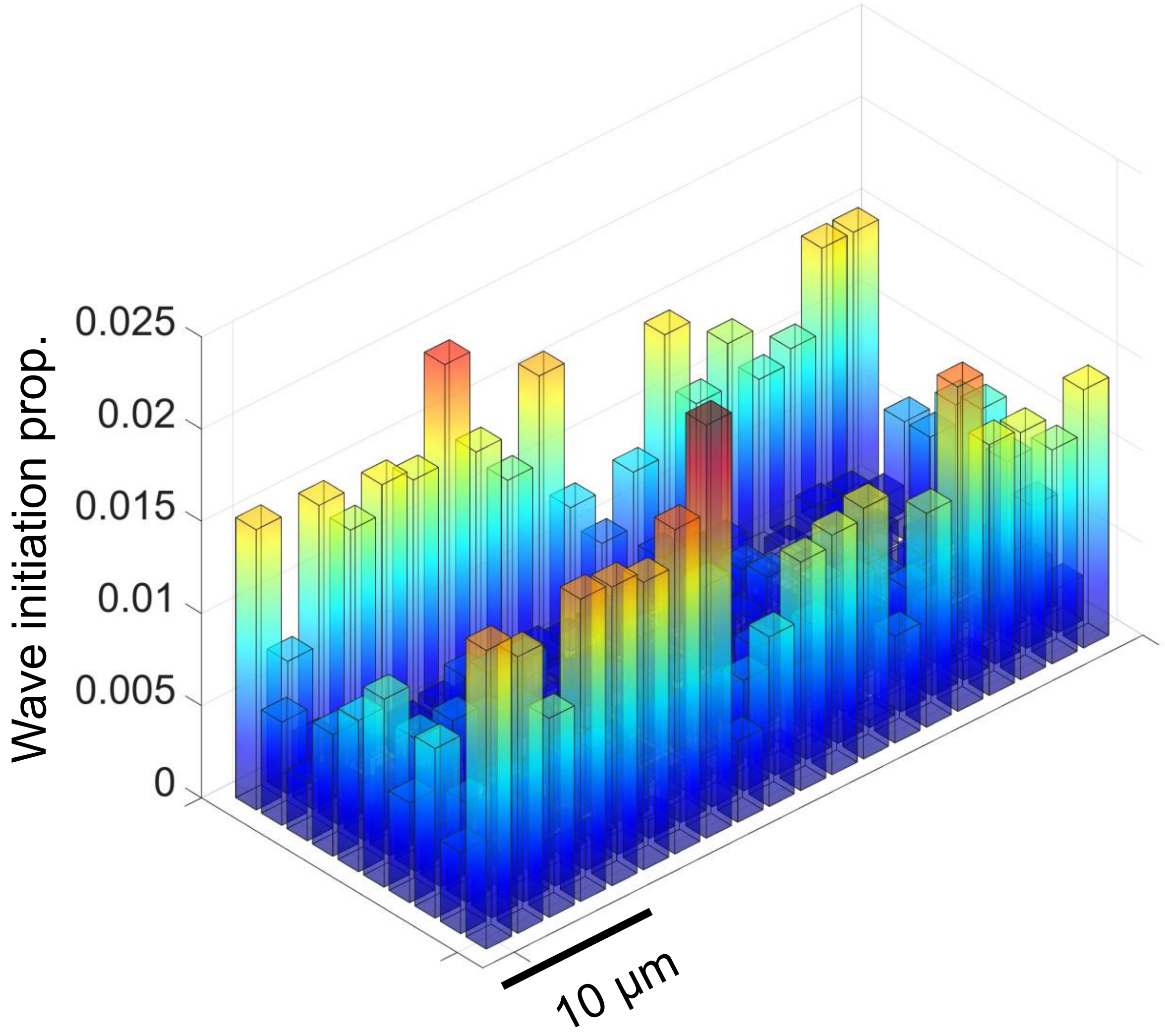

Figure S8. Ca^{2+} waves are predominantly initiated in proximity to the cell periphery. This three-dimensional bar graph illustrates the frequency of Ca^{2+} wave initiation points. Cell borders exhibit significantly higher frequencies (74% of waves originate within the outer two layers) compared to the central regions of the cell (26%), attributable to the limited sink effect near the cell periphery.

Figure S8
