# Supplementary material for: Gerrymandering in the heart: How tissue heterogeneities promote premature ventricular contractions
Source: Biophys J. 2026 Apr 6;125(9):2140–51. doi: 10.1016/j.bpj.2026.03.057 (PMC13351706; doi:10.1016/j.bpj.2026.03.057)
Supplement: Document S2. Article plus supplemental information [file mmc16.pdf]

Biophysical Journal
Article

# Gerrymandering in the heart: How tissue heterogeneities promote premature ventricular contractions

Daisuke Sato[1,*] and Donald M. Bers[1]
[1]Department of Pharmacology, University of California, Davis, Davis, CA 95616, USA

ABSTRACT Premature ventricular contractions (PVCs) are abnormal heartbeats that can trigger life-threatening arrhythmias. One mechanism responsible for PVCs is triggered activity. In this process, spontaneous calcium ($Ca^{2+}$) release from the sarcoplasmic reticulum and subsequent $Ca^{2+}$ waves at the subcellular level promote transient inward currents that lead to delayed afterdepolarizations at the cellular level. In uniform, healthy tissue, such events are often benign because the surrounding healthy cells act as an electrical sink, suppressing the abnormal signals from a few pathological source cells. This phenomenon is known as "source-sink mismatch." In this study, using computational modeling, we show how structural and functional tissue heterogeneities can overcome this protective mechanism. Regions of weak electrical coupling or specific cellular arrangements can electrically isolate small clusters of pathological cells. Within such isolated regions, the number of abnormal cells can more easily reach the "critical mass" required to overcome the source-sink mismatch and initiate a PVC. This principle, where the spatial arrangement of a minority group enables it to determine a local outcome of the whole group, is analogous to the political concept of gerrymandering. Our findings provide a novel mechanistic framework for understanding arrhythmia initiation and highlight the critical role of tissue heterogeneity.

SIGNIFICANCE This study reveals how spatial heterogeneities in the heart can trigger premature ventricular contractions (PVCs), a precursor to life-threatening arrhythmias. We show that the specific location of a small cluster of abnormal cells within regions of weak electrical coupling can overcome the protective source-sink mismatch to initiate a PVC. This phenomenon is governed by spatial statistics and electrotonic source-sink balance. It is analogous to how manipulating electoral district boundaries can influence election outcomes; this cardiac "gerrymandering" enables a local cluster of pathological cells to trigger an arrhythmia. These findings provide novel mechanistic insights into PVC formation and suggest therapeutic strategies could target regions of reduced electrical coupling to prevent arrhythmias, underscoring the critical role of tissue heterogeneity in cardiac electrophysiology.

## INTRODUCTION

Premature ventricular contractions (PVCs) are abnormal heartbeats that can lead to life-threatening ventricular tachycardia and ventricular fibrillation.[1–4] One mechanism responsible for PVCs is triggered activity. Under normal conditions, calcium ($Ca^{2+}$) sparks in cardiac myocytes are isolated, and spontaneous $Ca^{2+}$ waves rarely occur.[5,6] However, under pathological conditions such as a $Ca^{2+}$ overload or ryanodine receptor (RyR) sensitization, spontaneous $Ca^{2+}$ sparks and subsequent $Ca^{2+}$ waves during diastole lead to abnormal elevations of cytosolic $Ca^{2+}$ concentration ($[Ca^{2+}]_i$). This rise in $[Ca^{2+}]_i$ induces transient inward currents ($I_{ti}$) primarily through the sodium ($Na^+$)-$Ca^{2+}$ exchanger (NCX), which promotes delayed afterdepolarizations (DADs).[7–13] If this depolarization is sufficiently large to activate $Na^+$ channels, it triggers a new action potential (AP) at the cellular level.[14–19]

At the tissue level, however, $Ca^{2+}$ waves in just a few cells typically fail to trigger a PVC because the cells are electrotonically coupled by gap junctions, and the surrounding healthy cells act as a sink, keeping the membrane potential below the threshold for $Na^+$ channel activation.[20,21] This phenomenon is known as "source-sink mismatch".[21–24] We have previously shown that overcoming this "source-sink mismatch" in homogeneous tissue requires a large number

Submitted December 6, 2024, and accepted for publication March 27, 2026.
*Correspondence: dsato@ucdavis.edu
Editor: Manu Ben Johny.
https://doi.org/10.1016/j.bpj.2026.03.057
© 2026 The Author(s). Published by Elsevier Inc. on behalf of Biophysical Society.
This is an open access article under the CC BY license (http://creativecommons.org/licenses/by/4.0/).

of DAD-susceptible cells, on the order of $\sim 10^2$, $\sim 10^4$, and $\sim 10^6$ in one-, two-, and three-dimensional tissues, respectively.[21]

However, in reality, cardiac tissue is structurally and functionally heterogeneous.[1,25] Pathological remodeling associated with heart failure, myocardial infarction (MI), and hypertrophy introduces substantial structural and functional heterogeneity.[26,27] Fibrosis disrupts the normal arrangement of cardiomyocytes and introduces electrically insulating barriers, whereas gap junction remodeling reduces intercellular coupling and creates conduction block or slow conduction zones.[28–32] Fibroblasts and myofibroblasts can also electrically interact with myocytes and alter conduction properties.[31,32] Although these heterogeneous substrates are well-known to promote reentrant arrhythmias, their role in triggered arrhythmias such as PVCs is less well understood.

In this study, we hypothesize that the diverse forms of tissue heterogeneity alter the local source-sink balance and create conditions where a small number of DAD-susceptible cells can initiate PVCs. Specifically, localized regions of reduced electrical coupling or spatial arrangements of cells can electrically isolate a small cluster of pathological cells. Within such regions, the local balance of source and sink can be altered in favor of propagation, even if the global majority of tissue is composed of healthy cells. This statistical source-sink electrophysiological principle is analogous to the political concept of gerrymandering, a term coined after Massachusetts politician Elbridge Gerry, who in 1812 endorsed a strategy of redrawing electoral districts to benefit a particular party. Just as the spatial arrangement of a minority of voters can dictate an electoral outcome, we will show that the spatial arrangement of a minority of diseased cells can dictate electrical outcomes in cardiac tissue. This work provides new mechanistic insights into how pathological tissue remodeling lowers the threshold for triggered arrhythmias and underscores the importance of spatial heterogeneity in arrhythmogenesis.

## MATERIALS AND METHODS

In this study, we used physiologically detailed computational models of cardiac tissue to investigate how tissue heterogeneity affects the initiation of triggered arrhythmias. This computational approach allows for precise control over cellular properties and tissue architecture, enabling a systematic investigation into how structural and electrical heterogeneity can promote arrhythmogenesis. Figure 1A shows our models of a single cell, one-dimensional cable, and two-dimensional tissue.

### Cellular model

To simulate physiologically realistic $Ca^{2+}$ waves and APs, we used a modified version of the physiologically detailed rabbit ventricular myocyte model originally developed by Restrepo and Karma.[33–36] This model is based on the AP model by Mahajan et al.[37] combined with the subcellular $Ca^{2+}$ cycling model by Restrepo et al.[38] In each $Ca^{2+}$ release unit (CRU), there are five compartments for $Ca^{2+}$: cytosolic $Ca^{2+}$, submembrane $Ca^{2+}$, cleft space $Ca^{2+}$, network SR $Ca^{2+}$, and junctional SR $Ca^{2+}$. CRUs are coupled by $Ca^{2+}$ diffusion in the cytosol and the network SR. There are 19,305 CRUs (65 × 27 × 11) in the cell. Each CRU contains 100 RyRs, so there are 1,930,500 RyRs in the cell. The RyR channel, which is sensitive to both cytosolic $Ca^{2+}$ ($[Ca^{2+}]_i$) and SR $Ca^{2+}$ ($[Ca^{2+}]_{SR}$), is described by a four-state Markov model.

To simulate a proarrhythmic cellular phenotype, two modifications were made to create cells susceptible to DADs, hereafter termed "$DAD^+$" cells. First, the RyR sensitivity to $Ca^{2+}$ was increased to mimic conditions such as catecholaminergic polymorphic ventricular tachycardia mutations and heart failure. Second, to further promote spontaneous $Ca^{2+}$ wave activity, the maximum SERCA pump rate ($V_{max}$) was increased fourfold. In $DAD^+$ cells, spontaneous $Ca^{2+}$ waves occurred with a mean latency of 462 ms post pacing (SD 18 ms) and a mean peak $Ca^{2+}$ transient of 0.56 μM (SD 0.076 μM), indicating a stochastic but temporally localized process (Figure S1). Unmodified control cells, hereafter termed "$DAD^-$" cells, retained the original model parameters and did not exhibit spontaneous $Ca^{2+}$ waves.

### Tissue model

One-dimensional (1D) tissue was modeled as a linear cable of cells, and two-dimensional (2D) tissue was modeled as a rectangular grid of cells. In these models, individual cells were coupled diffusively through gap junctions. The ionic current flowing between any two cells ($I_{gap}$) is governed by Ohm's law, where the current is the product of the gap junction conductance ($G_{gap}$) and the difference in membrane potential between the cells:

$$I_{gap,i,j} = G_{gap}\left(V_i - V_j\right),$$

where $V_i$ and $V_j$ are the membrane potentials of the two cells, and $G_{gap}$ is the gap junctional conductance. For regions of normal coupling, $G_{gap}$ was set to 550 nS, consistent with values reported for well-coupled ventricular myocytes.[39] Regions of weak coupling were modeled by reducing $G_{gap}$ to 5.5 nS, and nonconductive barriers (e.g., scar tissue) were represented by setting $G_{gap} = 0$. By spatially varying the value of $G_{gap}$, the model effectively simulates the structural and electrical heterogeneity found in diseased hearts.

### Numerical implementation

Simulations were implemented in C/C++. Differential equations were solved using the forward Euler method with an adaptive time step, with a base step of 0.01 ms.

## RESULTS

### PVCs require a critical mass of proarrhythmic cells

The initiation of a PVC in cardiac tissue is governed by a "critical mass" principle. First, we consider the one-dimensional cable of five cells. The cable was paced sufficiently to reach steady state, and then the pacing was stopped. This protocol was used for all simulations in this study. The cable contains two cell types: normal cells where no $Ca^{2+}$ waves occur ($DAD^-$ cell) and DAD-susceptible cells where $Ca^{2+}$ waves occur readily when pacing is stopped ($DAD^+$ cell). Since cells are electrotonically well coupled ($G_{gap}$ = 550 nS), the membrane potentials are spatially averaged and nearly identical within this small length scale (0.6 mm) (Figures 1B and 1C voltage traces).

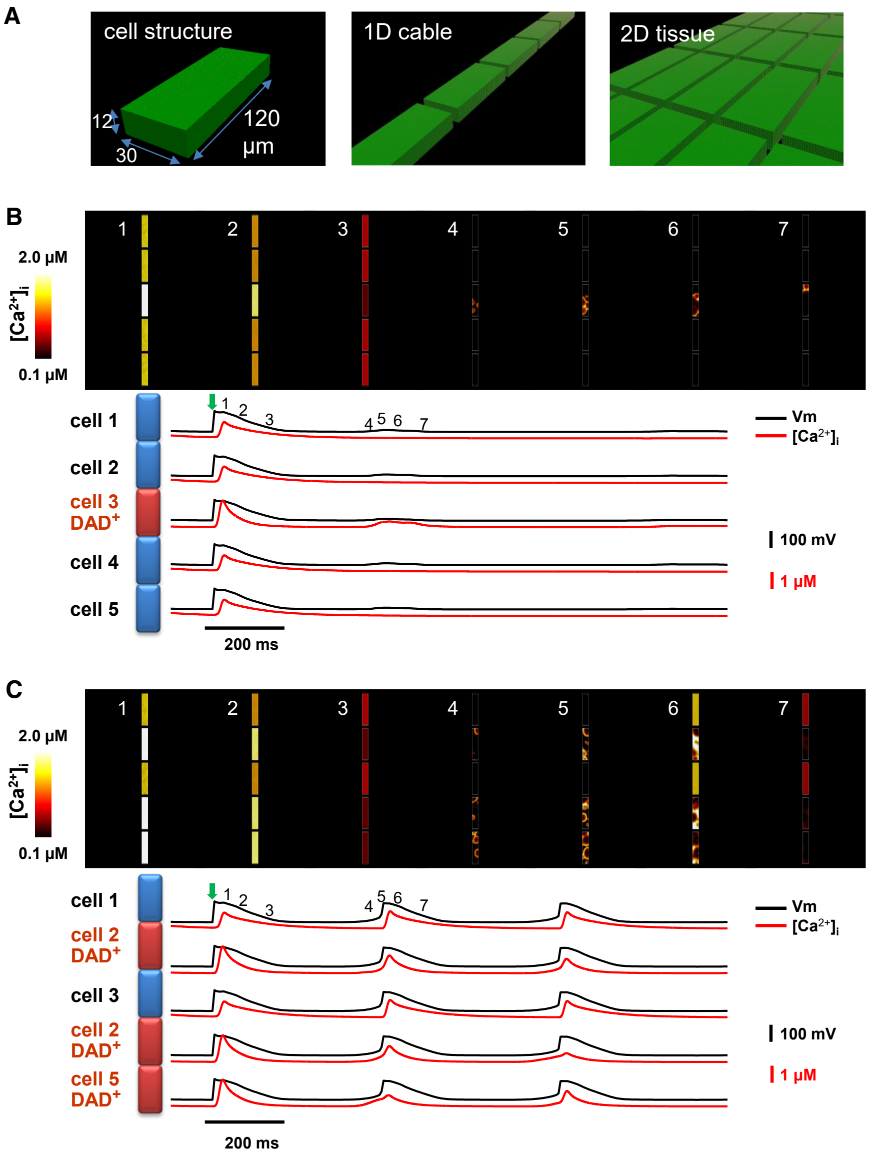


Figure 1 "Critical mass" is the basis for triggered activities. (A) cell model and tissue models. The size of the cell is 120 × 30 × 12 µm. In tissue, cells are coupled by gap junctions. (B) $Ca^{2+}$ waves in one cell (cell #3) in tissue do not cause triggered activity. Blue represents $DAD^{-}$ cells, and red represents $DAD^{+}$ cells. Top panels show snapshots of subcellular $[Ca^{2+}]_i$. Black line: membrane potential. Because the tissue size is very small, membrane potentials are almost identical. Red line: average $[Ca^{2+}]_i$. The tissue was paced until it reached the steady state. Green arrow indicates the last pacing beat (time = 0 ms) (see Video S1). The uncoupled case is shown in Figure S2A. (C) $Ca^{2+}$ waves must occur in at least three out of five cells (cells 2, 4, and 5) to overcome source-sink mismatch. Blue represents $DAD^{-}$ cells, and red represents $DAD^{+}$ cells (see Video S2). The uncoupled case is shown in Figure S2B.

Figure 1B shows that when $Ca^{2+}$ waves occurred in only one cell (cell 3), a small DAD was observed in the tissue (at time points 4–7, and most apparent in cell 3). However, this DAD was too small to initiate a new AP because the other four cells acted as a current sink, maintaining the membrane potential near the resting level. Only when $Ca^{2+}$ waves occurred in a sufficient number of cells to overcome this sink, the DAD became sufficiently larger for the $Na^{+}$ channel activation and caused PVCs (cells 2, 4, and 5 in Figure 1C). In this specific case, the minimum number of $DAD^{+}$ cells required to initiate a PVC was three. This minimum number is often called the "critical mass" and depends on various factors, including excitability of the cell, NCX density, SR $Ca^{2+}$ release flux, and timing of SR $Ca^{2+}$ releases.[20,21,40,41] For example, decreasing excitability by increasing $I_{K1}$ (Figure S3A) or decreasing $I_{Na}$ (Figure S3B) increases the minimum number of $DAD^{+}$ cells required for a PVC initiation. Conversely, increasing excitability by decreasing $I_{K1}$ (Figure S3C) or increasing $I_{Na}$ (Figure S3D) decreases the minimum number of $DAD^{+}$ cells required for a PVC initiation.

## PVC and gerrymandering analogy

Next, we consider a 2D tissue (5 × 5 cells, Figures 2A–2C) where nine of the 25 cells (36%) were $DAD^{+}$. Red circles in the left panels indicate the locations of $DAD^{+}$ cells. When all cells were homogeneously well coupled ($G_{gap}$ = 550 nS), no PVCs occurred because the $DAD^{+}$ fraction was below the critical mass for a PVC initiation, which was about 48% in this case (Figure 2A).

However, introducing electrical heterogeneity can promote PVCs. When the tissue was partitioned by a line

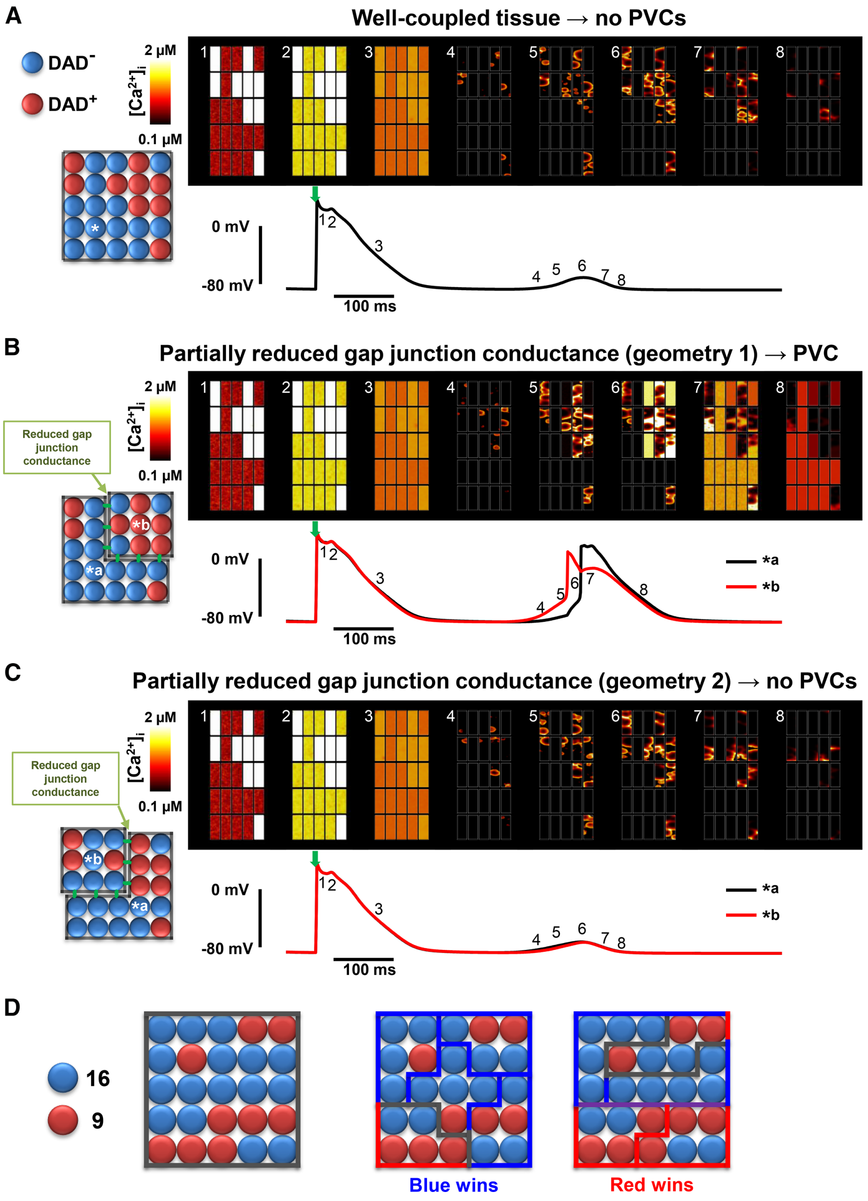


Figure 2 Gerrymandering and its effect on PVCs. (A) 5 × 5 tissue. Red circle in the left panel: $DAD^+$ cell. Blue circle in the left panel: $DAD^-$ cell. Nine out of 25 cells (36%) are $DAD^+$. In homogeneous tissue, $Ca^{2+}$ waves induce a DAD but cannot initiate a PVC. Top panels show snapshots of subcellular $[Ca^{2+}]_i$. The membrane potential was recorded from "*" in the left panel. Green arrow indicates the last pacing beat. Because cells are well coupled, membrane potentials are almost identical (see Video S3). (B) Gap junction conductance was reduced (*left panel*), and a PVC occurred. Membrane potentials were recorded from "*a" and "*b" in the left panel. 67% (above the threshold) of cells are $DAD^+$ in the top right corner, and 19% of cells are $DAD^+$ in the remaining part of the tissue. The membrane potential elevates in the top right corner first. Then, an AP wave starts and spreads to the remaining part of the tissue (see Video S4). (C) Gap junction conductance was reduced (*left panel*) in a different way. 33% of cells are $DAD^+$ in the top left corner, and 38% of cells are $DAD^+$ in the remaining part of the tissue. In this case, PVCs were not observed since $DAD^+$ cells could not overcome source-sink mismatch in both regions. (see Video S5) (D) Schematic illustration of gerrymandering. There are 16 blue dots and nine red dots. Depending on the shape of voting districts, results can be different.

of weak gap junction conductance ($G_{gap}$ = 5.5 nS), the electrical behavior of each subregion was primarily determined by its local cell population (Figure 2B). Although the global percentage of $DAD^+$ cells remained unchanged, this division created a subregion with a local, superthreshold majority of $DAD^+$ cells (six of nine cells, or 67%). This local majority generated a sufficiently large DAD that initiated an AP, which then propagated throughout the entire tissue (Figure 2B, panels 4–7). In contrast, a different partitioning geometry that did not create a superthreshold subregion failed to elicit PVCs (Figure 2C).

This phenomenon is analogous to the political concept of "gerrymandering," where the strategic drawing of boundaries determines outcomes (Figure 2D).[42] Similarly, in the formation of PVCs, reducing cell-to-cell coupling can electrically isolate a region of tissue, shifting the local source-sink balance. This demonstrates that the spatial arrangement of proarrhythmic cells is a critical determinant of a PVC initiation.

## The shorter space constant creates statistical hotspots for PVC initiation

The typical human heart size is much larger than the tissue we considered in Figure 2. The rise of the membrane voltage due to $Ca^{2+}$ waves quickly declines with increasing distance from the source cell. This electrotonic decay is characterized by the space constant, which defines the length scale over which the membrane voltage is

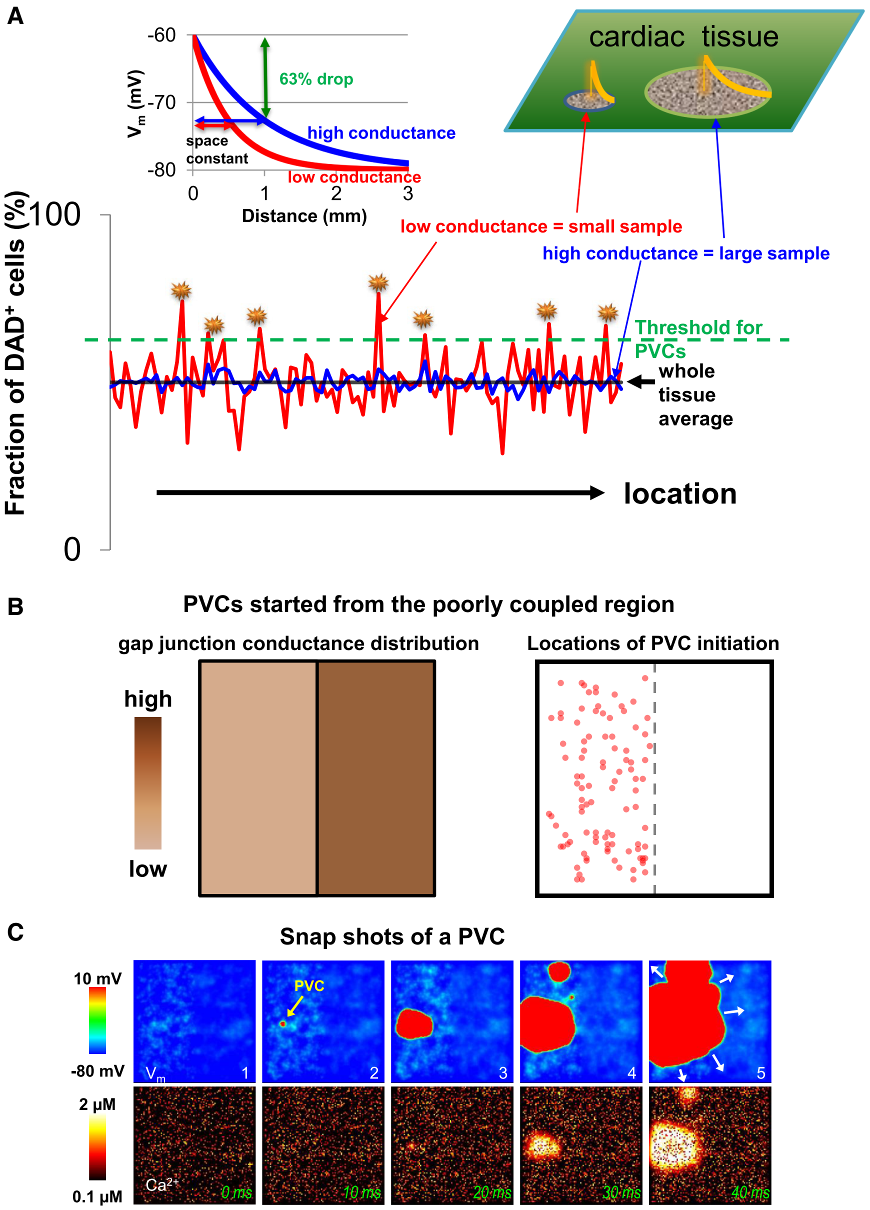


Figure 3 Space constant and PVCs. (A) Schematic illustration of how shorter space constant promotes local potential elevation. The distance that the membrane voltage drops to 36.8% of the source cell value is called a "space constant." If the space constant becomes smaller, the moving average fluctuates more. Blue line: large space constant. Red: small space constant. (B) 1.5 cm × 1.5 cm tissue. The tissue was bisected into two regions. The right side of the tissue has a higher gap junction conductance of 110 nS, whereas the left side has a lower gap junction conductance of 39.6 nS (*left panel*). The right panel indicates the locations of PVC initiation points from 100 simulations (different random number seeds). The left side (shorter space constant side) was the origin of all PVCs across all 100 simulations. Note that $Ca^{2+}$ waves occur uniformly randomly in tissue. (C) Top panels: snapshots of membrane potential. Bottom panels: snapshots of $[Ca^{2+}]_i$ (see Video S6).

averaged. The distance at which the membrane voltage drops to 36.8% ($=e^{-1}$) of the source cell value is called the "space constant" or "length constant" (Figure 3A).[43–45] The space constant depends on the strength of the electrotonic coupling through gap junctions. Normally, the space constant is 0.36–0.88 mm,[43] and the membrane voltage in tissue is averaged roughly within this range. The initiation of a PVC depends not on the global fraction of DAD⁺ cells, but on the number of DAD⁺ cells within a local region defined by the space constant.

As the gap junction conductance decreases, the number of cells within the range of the space constant decreases. This increases the statistical fluctuation of the DAD⁺ fraction in any given local area, making it more likely that a small region will randomly contain a supercritical mass of DAD⁺ cells. This fluctuation of the DAD⁺ fraction is proportional to the inverse of the square root of the number of cells within the range of the space constant. If DAD⁺ cells are uniformly randomly distributed in tissue, as the space constant decreases, the fraction of DAD⁺ cells within the range of the space constant can deviate substantially from the global tissue average, creating more chances to exceed the threshold for PVCs (Figure 3A).

To test this principle, we simulated a 2D tissue model (1.5 × 1.5 cm, 100 × 100 cells) composed of two distinct regions (Figures 3B and 3C). The right half of the tissue was modeled with high gap junction

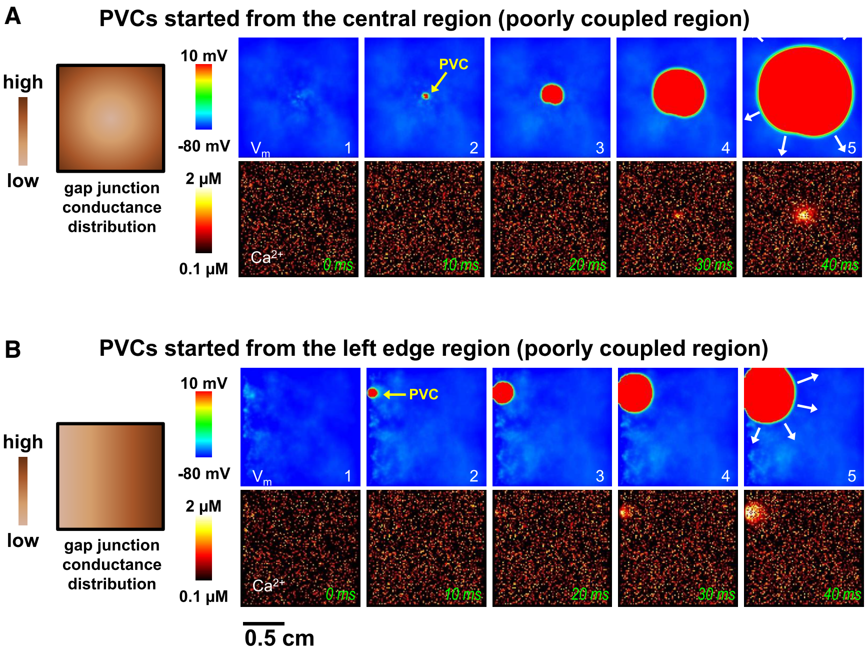


Figure 4 PVCs tend to start from poorly coupled regions. (A) 1.5 cm × 1.5 cm tissue. The highest gap junction conductance is 550 nS, and the lowest one is 5.5 nS. The center of tissue has the shortest space constant, and a PVC was initiated near the center. Note that $Ca^{2+}$ waves occur uniformly randomly in tissue. Top panels: snapshots of membrane potential. Bottom panels: snapshots of $[Ca^{2+}]_i$ (see Video S7). (B) 1.5 cm × 1.5 cm tissue. The left edge has the shortest space constant, and a PVC was initiated near the left edge. Top panels: snapshots of membrane potential. Bottom panels: snapshots of $[Ca^{2+}]_i$ (see Video S8). Additional simulations with different seeds of the random number generator are shown in Figure S2.

conductance ($G_{gap}$ = 110 nS) representing a large space constant, whereas the left half of the tissue was modeled with low conductance ($G_{gap}$ = 39.6 nS) representing a shorter space constant (Figure 3B, left panel). $DAD^+$ cells were distributed with a uniform random probability across the entire tissue. Despite the spatially uniform distribution of $DAD^+$ cells, every PVC (100 out of 100 simulations) originated from the left side of the tissue (the region with the shorter space constant) (Figure 3B, right panel). This demonstrates that this region is far more vulnerable to PVC initiation. Figure 3C shows snapshots from a representative simulation where $DAD^+$ cells in the poorly coupled region successfully raise the membrane potential to the $Na^+$ channel activation threshold, initiating a propagating PVC that spreads throughout the tissue.

We further simulated tissues with spatially continuous gradients of electrical heterogeneity. We simulated a tissue where gap junction conductance was lowest in the center (5.5 nS) and gradually increased toward the periphery (550 nS). PVCs consistently originated from the central region (Figure 4A). We also tested a different configuration where the shortest space constant was located along the left edge of the tissue. PVCs consistently originated from the left edge region (Figure 4B).

These results demonstrate the robust relationship between the site of poor coupling and the origin of PVCs (additional examples are in Figure S4, Videos S9, S10, and S11), and that the spatial pattern of electrical heterogeneity is a critical determinant of where focal arrhythmias will begin. The poorly coupled regions (Figures 3B and 3C) of tissue such as the infarct border zone effectively create focal "hotspots" that are highly susceptible to initiating a PVC, even when the underlying cellular triggers occur randomly anywhere in the tissue.

To further validate our hypothesis, we utilized a cellular automaton model, which is a variation of Conway's Game of Life.[46] In this model, each cell is either "alive" or "dead," and time progresses in discrete steps. The state of a cell is determined by the percentage of "alive" cells within its local neighborhood. If this percentage exceeds a predefined critical mass, the cell and its neighbors become "alive," analogous to a membrane voltage depolarization that reaches the excitation threshold. Conversely, if the critical mass is not met, the cells become "dead," analogous to a DAD that fails to trigger an action potential.

The simulations begin with a random distribution of "alive" and "dead" cells. As shown in Figure S5, this can lead to propagating waves of "alive" cells (Figure S5A) or scenarios where the entire grid becomes "dead" (Figure S5B). The space constant was modeled by varying the neighborhood size. A short space constant is represented by a nine-cell neighborhood (a cell plus its eight immediate neighbors) (Figure S5C), whereas a long space constant is represented by a 25-cell neighborhood (a cell plus its 24 surrounding neighbors) (Figure S5D).

To simulate the heterogeneous tissue explored in our physiologically detailed models, we implemented a 50 × 50 grid with two distinct rules. The left half of the grid utilized the nine-cell neighborhood (short space constant), whereas the right half utilized the 25-cell

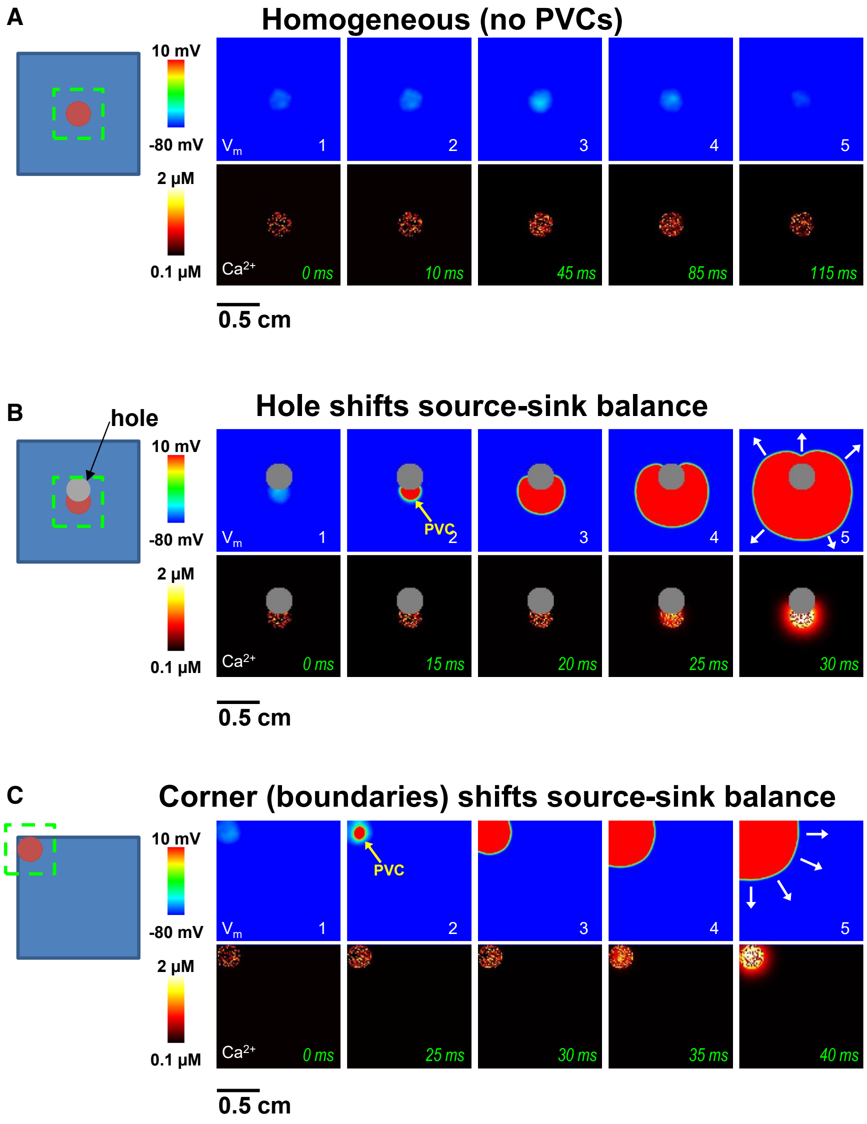


Figure 5 Tissue shapes affect PVCs formation. (A) Homogeneous 1.5 cm × 1.5 cm tissue (control). The red region in the left panel contains $DAD^+$ cells. Due to source-sink mismatch, only a DAD was observed (see Video S12). (B) The hole can promote PVCs. The simulation parameters are identical to those in Figure 5A except the hole. With the hole, the number of $DAD^+$ cells reached the critical mass within the green box. The hole plays the role of a blank vote (a neutral element in the local source-sink balance) (see Video S13). (C) When $DAD^+$ cells are close to the corner (or edge), PVCs occur readily. $DAD^+$ cells are surrounded by $DAD^-$ cells in every direction when $DAD^+$ cells are located at the center (Figure 5A). However, at the corner, $DAD^+$ cells are surrounded by $DAD^-$ cells only in two directions. The number of $DAD^+$ cells reached the critical mass within the green box (see Video S14).

neighborhood (long space constant). A sample simulation with an initial 10% density of "alive" cells is shown in Figure S6, where waves initiate on the left side, the region with the shorter space constant.

We quantified these dynamics by varying the initial density of "alive" cells (Figure S7). At a 5% initial density, waves rarely formed, but when they did, they exclusively initiated on the left side (Figure S7A). No waves were initiated on the right side within 10,000 trials. At a 10% density, wave initiation from the left side was common (>97%) (Figure S7B). Finally, at a 15% density, initiation from both sides became more frequent, but initiation still strongly favored the left side (Figure S7C). Initiation originating solely from the right side was exceedingly rare across all conditions. These results from the cellular automaton model are consistent with our primary finding in the physiologically detailed model.

## Tissue geometry alters the local source-sink balance

The geometric arrangement of cells and nonconductive obstacles can also modulate the local source-sink balance in favor of triggering an AP. In Figure 5A, $DAD^+$ cells are surrounded by $DAD^-$ cells in homogeneous tissue. Due to the strong sink effect of the surrounding $DAD^-$ cells, no PVCs were observed. However, if a nonconductive hole (representing scar tissue), which is neither a source nor a sink, is located near $DAD^+$ cells, a PVC starts because the hole shifts the local source-sink balance in favor of triggering an AP (Figure 5B, green box).

The same phenomenon occurred when $DAD^+$ cells were located at the edge or corner (Figure 5C). In homogeneous tissue (Figure 5A, green box), $DAD^+$ cells are surrounded

by DAD$^-$ cells in every direction. At the corner, DAD$^+$ cells are surrounded by DAD$^-$ cells only in two directions (Figure 5C, green box), which again shifts the local source-sink balance in favor of triggering an AP.

A key finding from our simulations is that wave initiation sites are not uniformly distributed, but instead, they show a strong preference for tissue boundaries, such as edges or nonconductive obstacles. This phenomenon of border-preferential initiation was statistically robust for membrane voltage waves in tissue. This principle also extends to the $Ca^{2+}$ wave initiation at the subcellular scale, where simulations show that spontaneous $Ca^{2+}$ waves likewise originate preferentially near border regions (Figure S8; 74% of waves originate within the outer two layers). These consistent findings at both the tissue and subcellular levels establish border-preferential initiation as a fundamental property of these excitable systems.

## DISCUSSION

Cardiac "gerrymandering" and the pathophysiological substrate.

In this study, we showed that tissue heterogeneities can enable a small, localized cluster of pathological cells to overcome the protective source-sink mismatch and initiate a focal arrhythmia. Under pathological conditions such as hypertrophy, heart failure, and MI, extensive structural and functional remodeling occurs.[25,26]

The fundamental requirement for PVCs is achieving a local critical mass of DAD$^+$ cells (Figure 1). This requirement is significantly altered by tissue heterogeneity (Figures 2, 3, and 4). The key finding is that the spatial arrangement of DAD$^+$ cells in relation to locations of weak electrical coupling or nonconductive barriers is a critical determinant of initiation of focal arrhythmia. By creating lines of reduced gap junction conductance, the tissue can be effectively "gerrymandered," electrically isolating a subregion where a local superthreshold majority of DAD$^+$ cells can emerge, even when the globally distributed population of DAD$^+$ cells is subthreshold (Figure 2). This local cluster can then generate a propagated AP that invades the rest of the tissue.

### The space constant determines hotspots for arrhythmia initiation

The critical mass principle works only within the range of the space constant (Figures 3 and 4). As the space constant becomes smaller, the critical mass is determined by a smaller number of cells. As the local population becomes smaller, the fraction of DAD$^+$ cells can deviate significantly from the global tissue average, and thus the chance to exceed the threshold for PVCs increases (Figure 3A). This statistical principle is evident in population data, where small towns often exhibit averages that deviate substantially from national averages simply due to their small sample size. Similarly, as gap junction coupling becomes weaker, the membrane potential is averaged from a smaller number of cells and can deviate significantly from the tissue average. Therefore, these regions tend to become initiation points of PVCs (Figures 3B, 4A, and 4B). Previously, we have shown that the number of DAD$^+$ cells required to form a PVC decreases when the gap junction conductance is reduced.[21] In that study, the gap junction conductance simply rescaled the system. In this study, the mechanism by which a small number of DAD$^+$ cells overcomes the source-sink mismatch is that gap junction conductance is reduced locally, thereby increasing fluctuations of the DAD$^+$ fraction within the range of the space constant. Therefore, regions with weak coupling are prone to becoming initiation points for PVCs.

### Impact of tissue geometry and boundaries

Geometric constraints, such as nonconductive holes or tissue boundaries, also reduce the local electrical sink, making it easier for a nearby cluster of DAD$^+$ cells to reach the critical mass for PVCs (Figure 5). These findings underscore that the spatial arrangement of pathological cells is as crucial as their absolute number or intrinsic electrophysiological properties for focal arrhythmias.

These observations can be generalized: any heterogeneity that alters the local source-sink balance to favor the source can promote PVCs.

### Implications for cardiac imaging and risk stratification

The principles demonstrated in this study provide a mechanistic framework for understanding how focal arrhythmia arise in clinically relevant disease states. In the heart, many factors such as gap junction remodeling,[28,30] fibroblasts,[31] and MI[29,47] reduce conduction and promote PVC formation so that a small number of DAD$^+$ cells can overcome the source-sink mismatch. For example, the regions of reduced gap junction conductance shown here occur in the peri-infarct border zone, a region of profound structural and electrical heterogeneity known to be prone to ventricular arrhythmias.[48,49] Our results provide a potential explanation for the clinical observation that the extent and complexity of the border zone are potent predictors of arrhythmic risk in postinfarction patients.

Similarly, the nonconductive holes and boundaries in the model represent the electrophysiological impact of dense collagenous scar tissue or areas of extensive interstitial fibrosis observed in both ischemic and nonischemic cardiomyopathies. These fibrotic regions act as passive obstacles that effectively reduce the number of healthy "sink" cells that a nearby cluster of "source" cells can depolarize to initiate a wave. The reduction of the "sink" at an edge or

obstacle offers a unifying mechanism that can explain how diverse pathologies, such as a focal MI scar and the diffuse fibrosis of heart failure, promote focal arrhythmias. It underscores that for the initiation of focal arrhythmias, the spatial architecture of the pathological substrate is as critical as the intrinsic properties of the cells themselves. Although the macroscopic appearance of these diseases is different, at the scale relevant to a small cluster of $DAD^+$ cells, their effects are functionally equivalent: they shift the local tissue to favor the source over the sink.

These findings have direct implications for the interpretation of advanced cardiac imaging.[50,51] The model suggests that clinical risk stratification should move beyond simply quantifying the total volume of scar or fibrosis. Instead, imaging biomarkers that capture the spatial architecture and heterogeneity of the pathological substrate are likely to be more powerful predictors of risk for triggered arrhythmias. Metrics such as border zone size, scar surface complexity, and textural analysis of fibrotic regions are more likely to identify proarrhythmic substrates than simple scar mass. Indeed, a large, compact, and well-defined scar may be less arrhythmogenic than a smaller, more fragmented scar with an extensive border zone, as the latter creates numerous small, isolated regions of tissue that are geometrically and electrically predisposed to focal arrhythmia initiation.

## Implications for therapeutic intervention

These findings provide a mechanistic framework that may more broadly help guide future therapeutic interventions for ventricular arrhythmias, particularly catheter ablation. Current ablation approaches typically focus on identifying and eliminating the precise site of trigger origin. However, when triggers arise from stochastic intracellular $Ca^{2+}$ events, the initiating cluster of cells is not fixed in space. The specific cells that reach the critical mass for propagation can vary from beat to beat, making the trigger location inherently dynamic. As a result, directly targeting the apparent focal origin may be challenging and may contribute to incomplete or nondurable outcomes.

Our results suggest an alternative conceptual approach. Arrhythmogenic risk in poorly coupled regions is determined not only by the presence of pathological cells but by the local structural architecture that reduces electrotonic averaging and lowers the effective critical mass threshold. In this framework, therapeutic success may depend less on eliminating every potential trigger and more on modifying the substrate that permits small pathological clusters to overcome source-sink balance. Targeted ablation or substrate homogenization aimed at regions of reduced coupling, sharp conductivity gradients, or geometries that favor local clustering could increase the effective sample size and restore protective electrotonic buffering. More broadly, imaging and mapping strategies that quantify heterogeneity architecture, rather than scar burden alone, may better identify high-risk regions for intervention.

## Generalization to 3D tissue and other excitable media

The space dimension is one of the geometrical factors. In 1D tissue, one cell has two neighbors. However, in 2D tissue, one cell has four to eight neighbors. In general, more $DAD^+$ cells are required in higher dimensional tissues. We previously showed that approximately $\sim 10^2$, $\sim 10^4$, and $\sim 10^6$ $DAD^+$ cells are required to trigger PVCs in homogeneous 1D, 2D, and 3D tissue, respectively.[21] In this study, we showed two examples of geometrical effects (effects of the hole in Figure 5B and the corner of the square tissue in Figure 5C). These observations can be generalized even in 3D tissue that geometry changes the source-sink balance within the range of the space constant. But more $DAD^+$ cells will be required for PVC initiation.

The principles of critical mass and spatial heterogeneity explored in this study are not limited to cardiac action potentials but are likely fundamental to a wide range of excitable media. For instance, the initiation of intracellular $Ca^{2+}$ waves requires a critical mass of several $Ca^{2+}$ sparks. In this study, we demonstrated border-preferential initiation of $Ca^{2+}$ waves (Figure S8). Dawson et al. made a model of $Ca^{2+}$ wave and called it the "fire-diffuse-fire" model, which is inspired by wildfire.[52] Moreover, the $Ca^{2+}$ spark itself requires multiple openings of RyR channels in the single cluster.[35] These phenomena are also found in other cell types such as HeLa cells and *Xenopus* oocytes.[53,54] Similar source-sink principles may be critical for seizure initiation in epilepsy. In that context, a localized focus of hyperexcitable neurons must overcome the inhibitory influence of the surrounding healthy tissue to trigger a large-scale, propagating seizure. These concepts even apply to collective human behavior. For example, Farkas et al. showed that 25–35 people are required to trigger a Mexican wave at the 1986 World Cup.[55,56] In these studies, heterogeneities were rarely considered. The general principles of local critical mass and spatial heterogeneity explored here are likely universal features of such excitable media.

## Study limitations

This study has several limitations. Our simulations were conducted in simplified 1D and 2D tissue models, which do not fully capture the complex 3D source-sink relationships of the intact ventricle, where the required critical mass of cells is substantially larger. Furthermore, our model considers only cardiomyocytes and does not include other cell types, such as fibroblasts, which are known to modulate electrical coupling and excitability in pathological states.

The specific proarrhythmic phenotype was based on a rabbit ventricular myocyte model, and although the principles of source-sink dynamics are general, the quantitative thresholds may differ in human cellular models. The modeled heterogeneities, such as nonconductive holes and linear regions of weak coupling, are idealizations of the complex, irregular structures found in diseased cardiac tissue. Although we use the gerrymandering analogy for its conceptual clarity, we acknowledge its limitations. Political gerrymandering is a static redrawing of boundaries, whereas the heart is a dynamic, excitable medium. Despite these differences and simplifications, the metaphor remains highly effective for illustrating how spatial architecture alone can determine a global outcome, and this study provides a robust conceptual framework for further understanding how spatial heterogeneity alters the local source-sink balance to promote focal arrhythmias.

Mathematical treatment of heterogeneities is nontrivial since there are infinite combinations of heterogeneities, and simplifications to find analytical solutions may lose fundamental properties of heterogeneous systems. Here, we demonstrated how to approach tissue heterogeneities for triggered arrhythmias, and many types of heterogeneities can be interpreted using the "critical mass" principle within the range of the space constant.

## CONCLUSION

In conclusion, this study provides a unified framework for understanding how various tissue heterogeneities contribute to arrhythmogenesis via a common mechanism: modulation of the local source-sink balance. Traditionally, research on triggered arrhythmogenesis has focused primarily on the intrinsic properties of the pathological "source" cells. However, our findings demonstrate that the properties of the surrounding "sink" tissue and the spatial arrangement of the source and sink are the critical factors in determining whether a focal event is suppressed or triggers a propagated arrhythmia. The concept of "cardiac gerrymandering" provides a novel and intuitive framework for understanding how various pathological tissue heterogeneities can contribute to arrhythmogenesis through this common mechanism. This work underscores that for the initiation of focal arrhythmias, "where" a pathological cell resides within the cardiac tissue is as important as "what" that cell is. This perspective has profound implications for understanding disease mechanisms, interpreting advanced cardiac imaging for risk stratification, and developing future therapeutic strategies to prevent sudden cardiac death.

## DATA AVAILABILITY

The authors confirm that the data supporting the findings of this study are available within the article. The simulation code is available on GitHub or upon request.

## ACKNOWLEDGMENTS

Supported by NIH grants (P01-HL141084, R01HL142282 to D.M.B.; R01HL149349 to D.S. and D.M.B.).

## AUTHOR CONTRIBUTIONS

D.S. performed mathematical modeling and computer simulations. D.S processed and analyzed the simulation data. D.S. and D.M.B. interpreted the data and wrote the manuscript. All authors approved the final version of the manuscript.

## DECLARATION OF INTERESTS

The authors declare no conflict of interests.

## SUPPLEMENTAL INFORMATION

Supplemental information can be found online at https://doi.org/10.1016/j.bpj.2026.03.057.

## REFERENCES


1. Roberts-Thomson, K. C., D. H. Lau, and P. Sanders. 2011. The diagnosis and management of ventricular arrhythmias. *Nat. Rev. Cardiol.* 8:311–321. https://doi.org/10.1038/nrcardio.2011.15.
2. Ng, G. A. 2006. Treating patients with ventricular ectopic beats. *Heart.* 92:1707–1712. https://doi.org/10.1136/hrt.2005.067843.
3. Callans, D. J. 2017. Premature Ventricular Contraction-induced Cardiomyopathy. *Arrhythm Electrophysiol Rev.* 6:153–155. https://doi.org/10.15420/aer.2017/6.4/EO1.
4. Latchamsetty, R., and F. Bogun. 2019. Premature Ventricular Complex–Induced Cardiomyopathy. *JACC (J. Am. Coll. Cardiol.): Clinical Electrophysiology.* 5:537–550. https://doi.org/10.1016/j.jacep.2019.03.013.
5. Cheng, H., M. R. Lederer, …, M. B. Cannell. 1996. Calcium sparks and [Ca2+]i waves in cardiac myocytes. *The American journal of physiology.* 270:C148–C159.
6. Bers, D. M. 2002. Cardiac excitation-contraction coupling. *Nature.* 415:198–205. https://doi.org/10.1038/415198a.
7. Lipp, P., and L. Pott. 1988. Transient inward current in guinea-pig atrial myocytes reflects a change of sodium-calcium exchange current. *J Physiol.* 397:601–630.
8. Tseng, G. N., and A. L. Wit. 1987. Characteristics of a transient inward current that causes delayed afterdepolarizations in atrial cells of the canine coronary sinus. *J. Mol. Cell. Cardiol.* 19:1105–1119.
9. January, C. T., and H. A. Fozzard. 1988. Delayed afterdepolarizations in heart muscle: mechanisms and relevance. *Pharmacol. Rev.* 40:219–227.
10. Verkerk, A. O., M. W. Veldkamp, …, J. H. Ravesloot. 2001. Ionic mechanism of delayed afterdepolarizations in ventricular cells isolated from human end-stage failing hearts. *Circulation.* 104:2728–2733.
11. Voigt, N., N. Li, …, D. Dobrev. 2012. Enhanced sarcoplasmic reticulum Ca2+ leak and increased Na+-Ca2+ exchanger function underlie delayed afterdepolarizations in patients with chronic atrial fibrillation. *Circulation.* 125:2059–2070. https://doi.org/10.1161/CIRCULATIONAHA.111.067306.
12. Schlotthauer, K., and D. M. Bers. 2000. Sarcoplasmic reticulum Ca(2+) release causes myocyte depolarization. Underlying mechanism and threshold for triggered action potentials. *Circ. Res.* 87:774–780.

13. Kistamás, K., R. Veress, …, D. A. Eisner. 2020. Calcium Handling Defects and Cardiac Arrhythmia Syndromes. *Front. Pharmacol.* 11:72. https://doi.org/10.3389/fphar.2020.00072.

14. Pogwizd, S. M., and D. M. Bers. 2004. Cellular basis of triggered arrhythmias in heart failure. *Trends Cardiovasc. Med.* 14:61–66. https://doi.org/10.1016/j.tcm.2003.12.002.

15. Priori, S. G., and P. B. Corr. 1990. Mechanisms underlying early and delayed afterdepolarizations induced by catecholamines. *Am. J. Physiol. Heart Circ. Physiol.* 258:H1796–H1805.

16. Pogwizd, S. M., and D. M. Bers. 2004. Cellular Basis of Triggered Arrhythmias in Heart Failure. *Trends Cardiovasc. Med.* 14:61–66. https://doi.org/10.1016/j.tcm.2003.12.002.

17. Sipido, K. R., V. Bito, …, M. A. Vos. 2007. Na/Ca Exchange and Cardiac Ventricular Arrhythmias. *Ann. N. Y. Acad. Sci.* 1099:339–348. https://doi.org/10.1196/annals.1387.066.

18. Katra, R. P., and K. R. Laurita. 2005. Cellular Mechanism of Calcium-Mediated Triggered Activity in the Heart. *Circ. Res.* 96:535–542. https://doi.org/10.1161/01.RES.0000159387.00749.3c.

19. Györke, S. 2009. Molecular basis of catecholaminergic polymorphic ventricular tachycardia. *Heart Rhythm.* 6:123–129. https://doi.org/10.1016/j.hrthm.2008.09.013.

20. Fujiwara, K., H. Tanaka, …, T. Takamatsu. 2008. Burst emergence of intracellular Ca2+ waves evokes arrhythmogenic oscillatory depolarization via the Na+-Ca2+ exchanger: simultaneous confocal recording of membrane potential and intracellular Ca2+ in the heart. *Circ. Res.* 103:509–518. https://doi.org/10.1161/CIRCRESAHA.108.176677.

21. Xie, Y., D. Sato, …, J. N. Weiss. 2010. So little source, so much sink: requirements for afterdepolarizations to propagate in tissue. *Biophys. J.* 99:1408–1415. https://doi.org/10.1016/j.bpj.2010.06.042.

22. Rohr, S., J. P. Kucera, …, A. G. Kleber. 1997. Paradoxical improvement of impulse conduction in cardiac tissue by partial cellular uncoupling. *Science.* 275:841–844.

23. London, B. 2017. United We Stand; Divided We Fibrillate? *Circ. Res.* 121:1302–1303. https://doi.org/10.1161/circresaha.117.312176.

24. Ciaccio, E. J., J. Coromilas, …, H. Garan. 2018. Source-Sink Mismatch Causing Functional Conduction Block in Re-Entrant Ventricular Tachycardia. *JACC Clin Electrophysiol.* 4:1–16. https://doi.org/10.1016/j.jacep.2017.08.019.

25. Burchfield, J. S., M. Xie, and J. A. Hill. 2013. Pathological Ventricular Remodeling: Mechanisms: Part 1 of 2. *Circulation.* 128:388–400. https://doi.org/10.1161/circulationaha.113.001878.

26. Burchfield, J. S., M. Xie, and J. A. Hill. 2013. Pathological Ventricular Remodeling. *Circulation.* 128:388–400. https://doi.org/10.1161/CIRCULATIONAHA.113.001878.

27. Pfeffer, M. A., and E. Braunwald. 1990. Ventricular remodeling after myocardial infarction. Experimental observations and clinical implications. *Circulation.* 81:1161–1172. https://doi.org/10.1161/01.cir.81.4.1161.

28. Lang, D., D. Sato, …, D. M. Bers. 2017. Calcium-Dependent Arrhythmogenic Foci Created by Weakly Coupled Myocytes in the Failing Heart. *Circ. Res.* 121:1379–1391. https://doi.org/10.1161/circresaha.117.312050.

29. de Bakker, J. M., F. J. van Capelle, …, J. R. Lahpor. 1993. Slow conduction in the infarcted human heart. 'Zigzag' course of activation. *Circulation.* 88:915–926. https://doi.org/10.1161/01.cir.88.3.915.

30. Kostin, S., M. Rieger, …, J. Schaper. 2003. Gap junction remodeling and altered connexin43 expression in the failing human heart. *In* Developments in Molecular and Cellular Biochemistry, Cardiac Cell Biology. E. Kardami, L. Hryshko, and N. Mesaeli, eds Springer US, pp. 135–144.

31. Miragoli, M., G. Gaudesius, and S. Rohr. 2006. Electrotonic Modulation of Cardiac Impulse Conduction by Myofibroblasts. *Circ. Res.* 98:801–810. https://doi.org/10.1161/01.RES.0000214537.44195.a3.

32. Nguyen, T. P., Z. Qu, and J. N. Weiss. 2014. Cardiac fibrosis and arrhythmogenesis: the road to repair is paved with perils. *J. Mol. Cell. Cardiol.* 70:83–91. https://doi.org/10.1016/j.yjmcc.2013.10.018.

33. Restrepo, J. G., and A. Karma. 2009. Spatiotemporal intracellular calcium dynamics during cardiac alternans. *Chaos.* 19:037115. https://doi.org/10.1063/1.3207835.

34. Sato, D., T. R. Shannon, and D. M. Bers. 2016. Sarcoplasmic Reticulum Structure and Functional Properties that Promote Long-Lasting Calcium Sparks. *Biophys. J.* 110:382–390. https://doi.org/10.1016/j.bpj.2015.12.009.

35. Sato, D., and D. M. Bers. 2011. How does stochastic ryanodine receptor-mediated Ca leak fail to initiate a Ca spark? *Biophys. J.* 101:2370–2379. https://doi.org/10.1016/j.bpj.2011.10.017.

36. Sato, D., S. Despa, and D. M. Bers. 2012. Can the sodium-calcium exchanger initiate or suppress calcium sparks in cardiac myocytes? *Biophys. J.* 102:L31–L33. https://doi.org/10.1016/j.bpj.2012.03.051.

37. Mahajan, A., Y. Shiferaw, …, J. N. Weiss. 2008. A rabbit ventricular action potential model replicating cardiac dynamics at rapid heart rates. *Biophys. J.* 94:392–410. https://doi.org/10.1529/biophysj.106.98160.

38. Restrepo, J. G., J. N. Weiss, and A. Karma. 2008. Calsequestrin-mediated mechanism for cellular calcium transient alternans. *Biophys. J.* 95:3767–3789. https://doi.org/10.1529/biophysj.108.130419.

39. Yao, J. A., D. E. Gutstein, …, A. L. Wit. 2003. Cell coupling between ventricular myocyte pairs from connexin43-deficient murine hearts. *Circ. Res.* 93:736–743. https://doi.org/10.1161/01.Res.0000095977.66660.86.

40. Myles, R. C., L. Wang, …, C. M. Ripplinger. 2012. Local beta-adrenergic stimulation overcomes source-sink mismatch to generate focal arrhythmia. *Circ. Res.* 110:1454–1464. https://doi.org/10.1161/CIRCRESAHA.111.262345.

41. Wasserstrom, J. A., Y. Shiferaw, …, G. L. Aistrup. 2010. Variability in timing of spontaneous calcium release in the intact rat heart is determined by the time course of sarcoplasmic reticulum calcium load. *Circ. Res.* 107:1117–1126. https://doi.org/10.1161/CIRCRESAHA.110.229294.

42. Martis, K. C. 2008. The original gerrymander. *Polit. Geogr.* 27:833–839.

43. Sfyris, G., N. Sperelakis, …, E. Stepp. 1993. Length constant as a function of intercellular coupling of a cardiac muscle strand analog with applied longitudinal current. *Math. Comput. Model.* 17:131–139. https://doi.org/10.1016/0895-7177(93)90097-I.

44. Weidmann, S. 1970. Electrical constants of trabecular muscle from mammalian heart. *J. Physiol.* 210:1041–1054. https://doi.org/10.1113/jphysiol.1970.sp009256.

45. Kléber, A. G., and C. B. Riegger. 1987. Electrical constants of arterially perfused rabbit papillary muscle. *J. Physiol.* 385:307–324. https://doi.org/10.1113/jphysiol.1987.sp016495.

46. Gardner, M. 1970. MATHEMATICAL GAMES. *Sci. Am.* 223:120–123.

47. Campos, F. O., Y. Shiferaw, …, M. J. Bishop. 2018. Microscopic Isthmuses and Fibrosis Within the Border Zone of Infarcted Hearts Promote Calcium-Mediated Ectopy and Conduction Block. *Front. Phys.* 6:2018. https://doi.org/10.3389/fphy.2018.00057.

48. Haghbayan, H., N. Lougheed, …, A. T. Yan. 2019. Peri-Infarct Quantification by Cardiac Magnetic Resonance to Predict Outcomes in Ischemic Cardiomyopathy. *Circ., Cardiovasc. Imaging.* 12:e009156. https://doi.org/10.1161/CIRCIMAGING.119.009156.

49. Rubenstein, J. C., D. C. Lee, …, J. J. Goldberger. 2013. A comparison of cardiac magnetic resonance imaging peri-infarct border zone quantification strategies for the prediction of ventricular tachyarrhythmia inducibility. *Cardiol. J.* 20:68–77. https://doi.org/10.5603/CJ.2013.0011.

50. Xie, E., E. Sung, …, J. Chrispin. 2022. Advanced imaging for risk stratification for ventricular arrhythmias and sudden cardiac death. *Front. Cardiovasc. Med.* 9 – 2022. https://doi.org/10.3389/fcvm.2022.884767.

51. Zeidan-Shwiri, T., Y. Yang, …, E. Crystal. 2015. Magnetic resonance estimates of the extent and heterogeneity of scar tissue in ICD patients with ischemic cardiomyopathy predict ventricular arrhythmia. *Heart Rhythm.* 12:802–808. https://doi.org/10.1016/j.hrthm.2015.01.007.

52. Dawson, S. P., J. Keizer, and J. E. Pearson. 1999. Fire–diffuse–fire model of dynamics of intracellular calcium waves. *Proc. Natl. Acad. Sci. USA*. 96:6060–6063. https://doi.org/10.1073/pnas.96.11.6060.

53. Bootman, M., E. Niggli, …, P. Lipp. 1997. Imaging the hierarchical Ca2+ signalling system in HeLa cells. *J. Physiol.* 499:307–314.

54. Berridge, M. J., P. Lipp, and M. D. Bootman. 2000. The versatility and universality of calcium signalling. *Nat. Rev. Mol. Cell Biol.* 1:11–21.

55. Farkas, I., D. Helbing, and T. Vicsek. 2002. Mexican waves in an excitable medium. *Nature.* 419:131–132. https://doi.org/10.1038/419131a.

56. Farkas, I., D. Helbing, and T. Vicsek. 2003. Human waves in stadiums. *Phys. Stat. Mech. Appl.* 330:18–24. https://doi.org/10.1016/j.physa.2003.08.014.

**Biophysical Journal, Volume *125***

## Supplemental information

# Gerrymandering in the heart: How tissue heterogeneities promote premature ventricular contractions

**Daisuke Sato and Donald M. Bers**

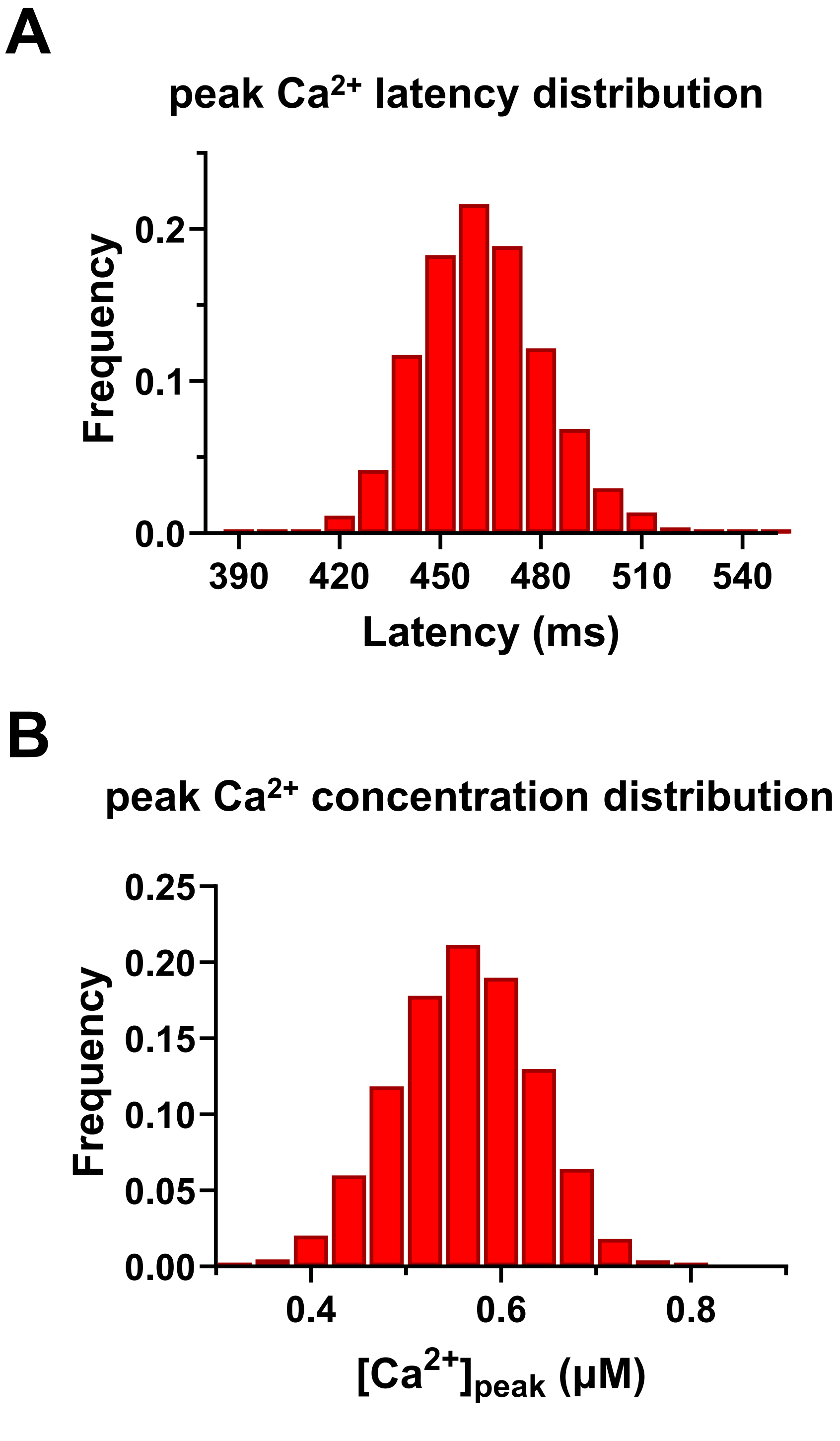


**Figure S1. Characteristics of spontaneous $Ca^{2+}$ waves.** (A) The histogram of the latency of the peak $[Ca^{2+}]_i$ from the preceding pacing. The mean latency is 461.9 ms and the standard deviation is 17.5 ms. (B) The histogram of the peak $Ca^{2+}$ transient amplitude. The mean peak $Ca^{2+}$ transient is 0.56 μM and the standard deviation is 0.076 μM. These histograms were obtained from 300 simulations of $DAD^{+}$ cells.

Figure S1

# Uncoupled tissue

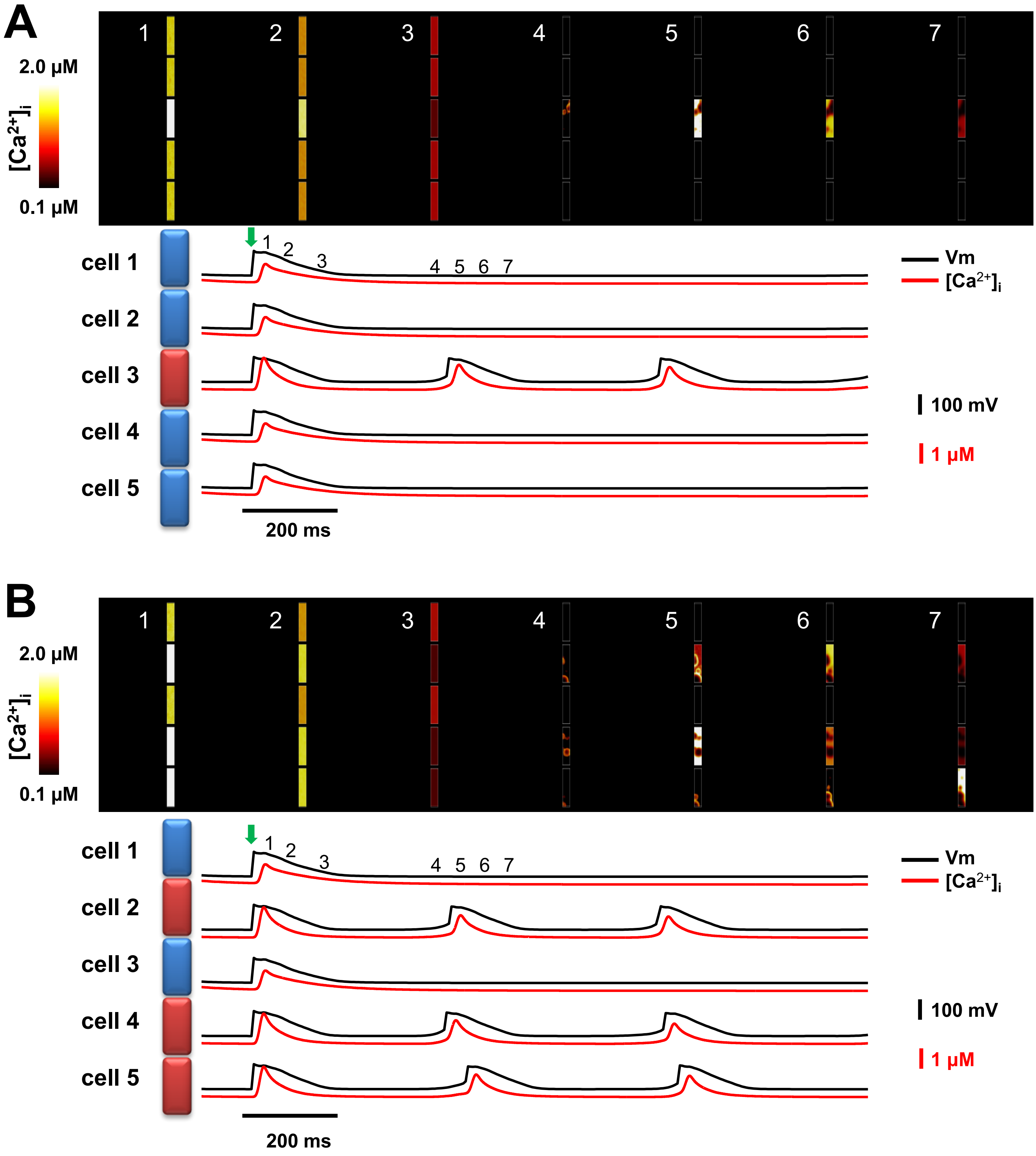


**Figure S2. The uncoupled cases corresponding to Figures 1B and 1C. (A)** The uncoupled case corresponding to Figure 1B. **(B)** The uncoupled case corresponding to Figure 1C.

**Figure S2**

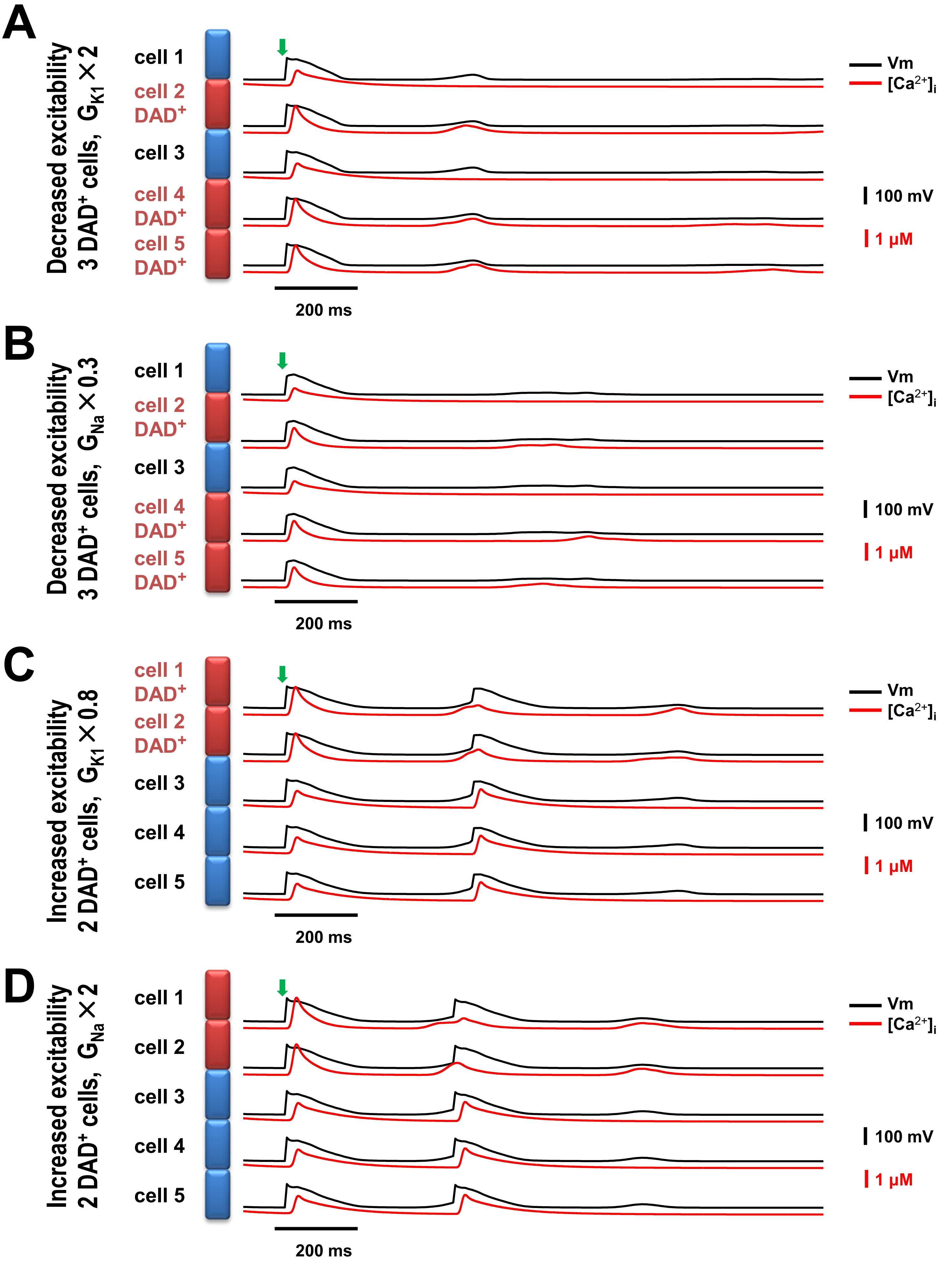
A
Decreased excitability
3 DAD+ cells, GK1 × 2
cell 1
cell 2
DAD+
cell 3
cell 4
DAD+
cell 5
DAD+
Vm
[Ca2+]i
100 mV
1 μM
200 ms
B
Decreased excitability
3 DAD+ cells, GNa × 0.3
cell 1
cell 2
DAD+
cell 3
cell 4
DAD+
cell 5
DAD+
Vm
[Ca2+]i
100 mV
1 μM
200 ms
C
Increased excitability
2 DAD+ cells, GK1 × 0.8
cell 1
DAD+
cell 2
DAD+
cell 3
cell 4
cell 5
Vm
[Ca2+]i
100 mV
1 μM
200 ms
D
Increased excitability
2 DAD+ cells, GNa × 2
cell 1
cell 2
cell 3
cell 4
cell 5
Vm
[Ca2+]i
100 mV
1 μM
200 ms

**Figure S3**

**Figure S3. Cellular excitability modulates the critical mass required for a PVC.** A reduction in cellular excitability increases the required number of $DAD^+$ cells for the initiation of a PVC. Conversely, an increase in cellular excitability reduces the required number of $DAD^+$ cells for a PVC. (A) Increasing $I_{K1}$ current ($\times$ 2) to decrease excitability prevented PVCs, even with three $DAD^+$ cells. (B) Decreasing $I_{Na}$ current ($\times$ 0.3) similarly prevented PVCs, even with three $DAD^+$ cells. (C) Decreasing $I_{K1}$ current ($\times$ 0.3) to enhance excitability led to a PVC with only two $DAD^+$ cells. (D) An increase in $I_{Na}$ ($\times$ 2) current also led to a PVC with two $DAD^+$ cells.

**Figure S3**

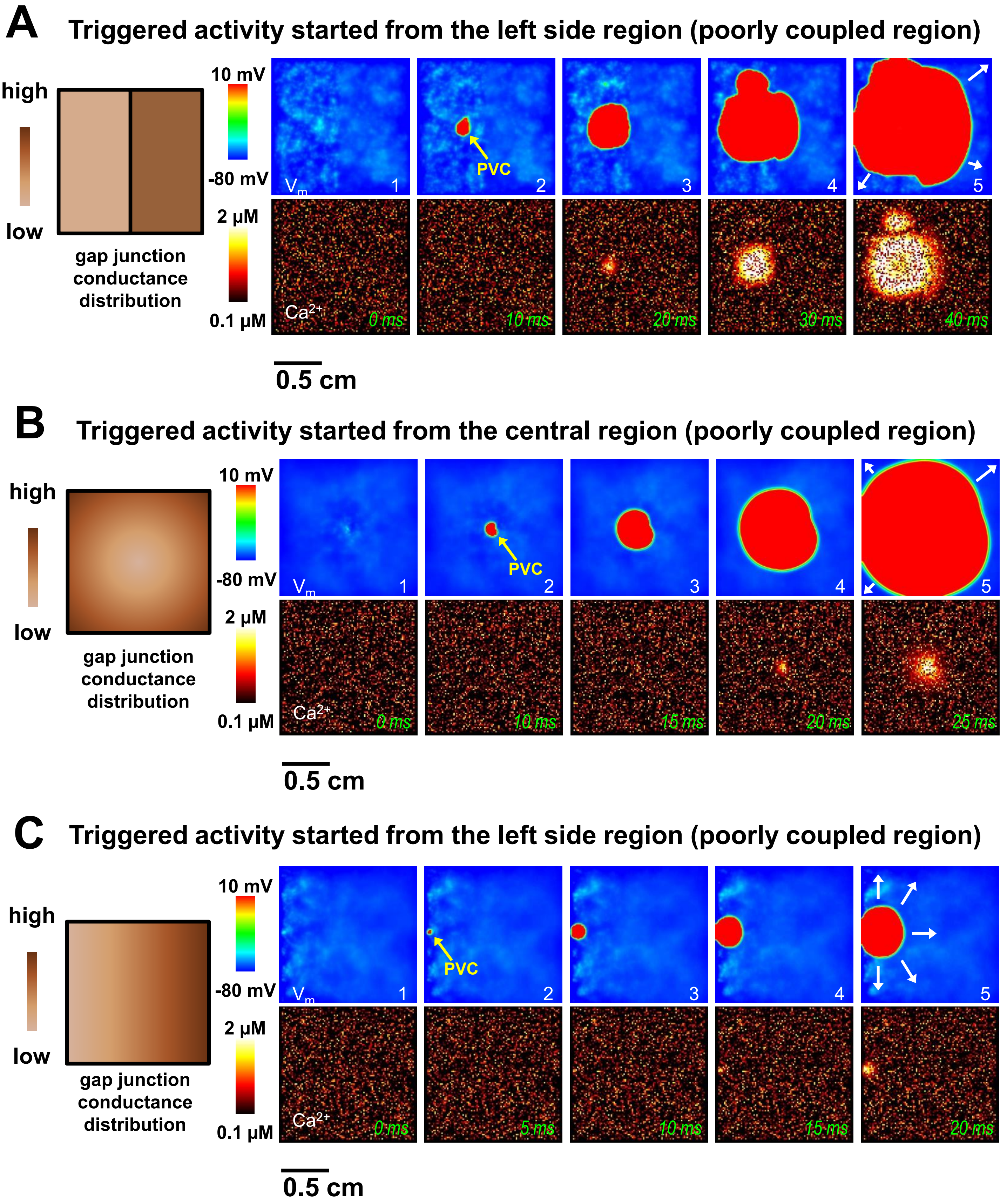


**Figure S4. Additional simulations with different seeds of the random number generator for Figures 3B, 4A and 4B. (A)** Simulation corresponding to Figure 3B. (see Supplementary Video S9) **(B)** Simulation corresponding to Figure 4A. (see Supplementary Video S10) **(C)** Simulation corresponding to Figure 4B. (see Supplementary Video S11)

**Figure S4**

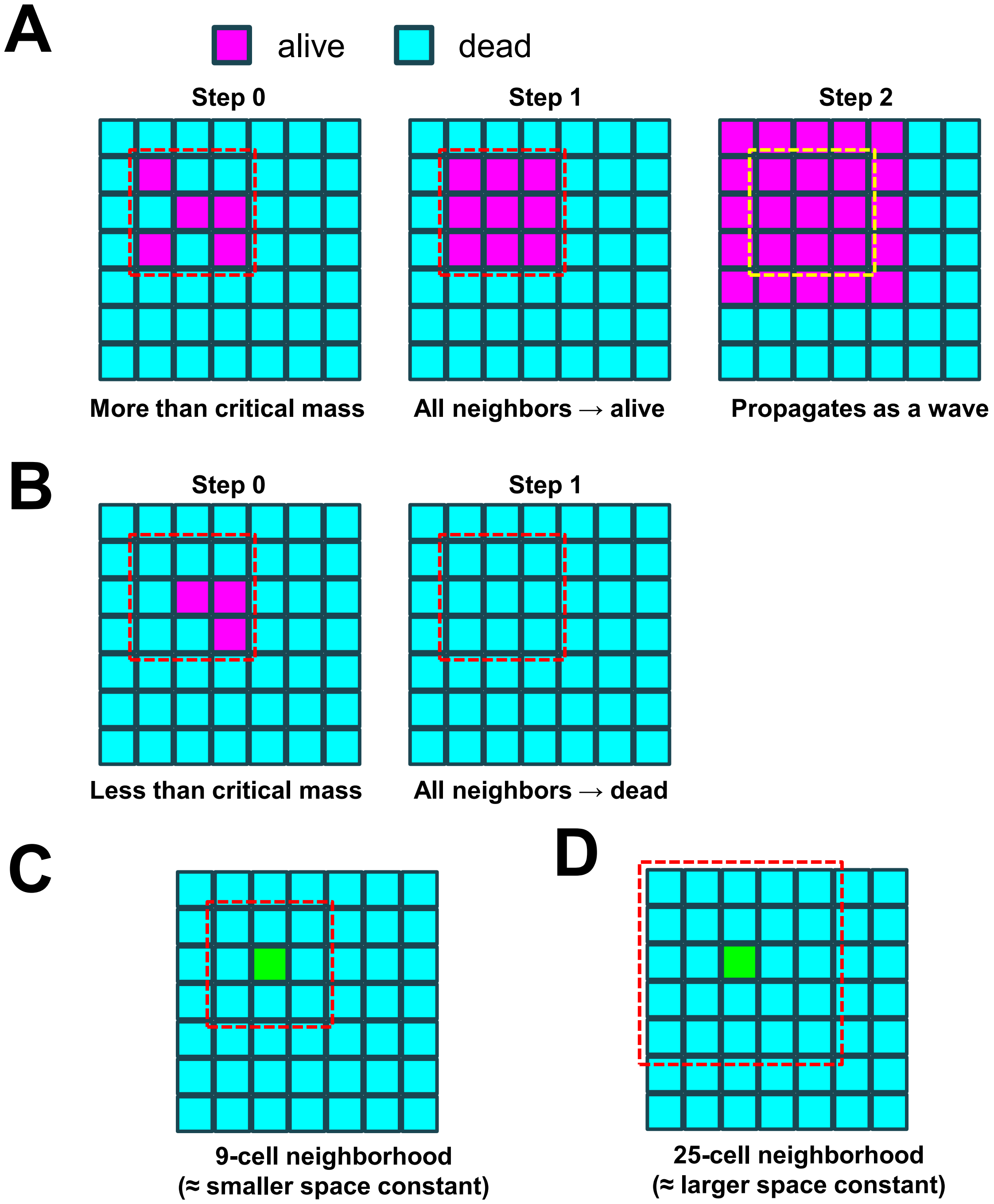


**Figure S5. Cellular automaton model illustrating how the space constant influences PVC initiation.** Magenta cells are "alive" and cyan cells are "dead." At each time step, a cell's state is determined by the percentage of "alive" cells in its local neighborhood (including itself). **(A)** If this percentage exceeds the 40% critical mass, all cells in the neighborhood become "alive." **(B)** Otherwise, they become "dead." **(C)** A 9-cell neighborhood (a cell plus its 8 immediate neighbors), representing a short space constant, meets the threshold if 4 or more cells are "alive" (4/9 ≈ 44%). A 25-cell neighborhood (a cell plus its 24 neighbors), representing a long space constant, meets the threshold if 10 or more cells are "alive" (10/25 = 40%).

**Figure S5**

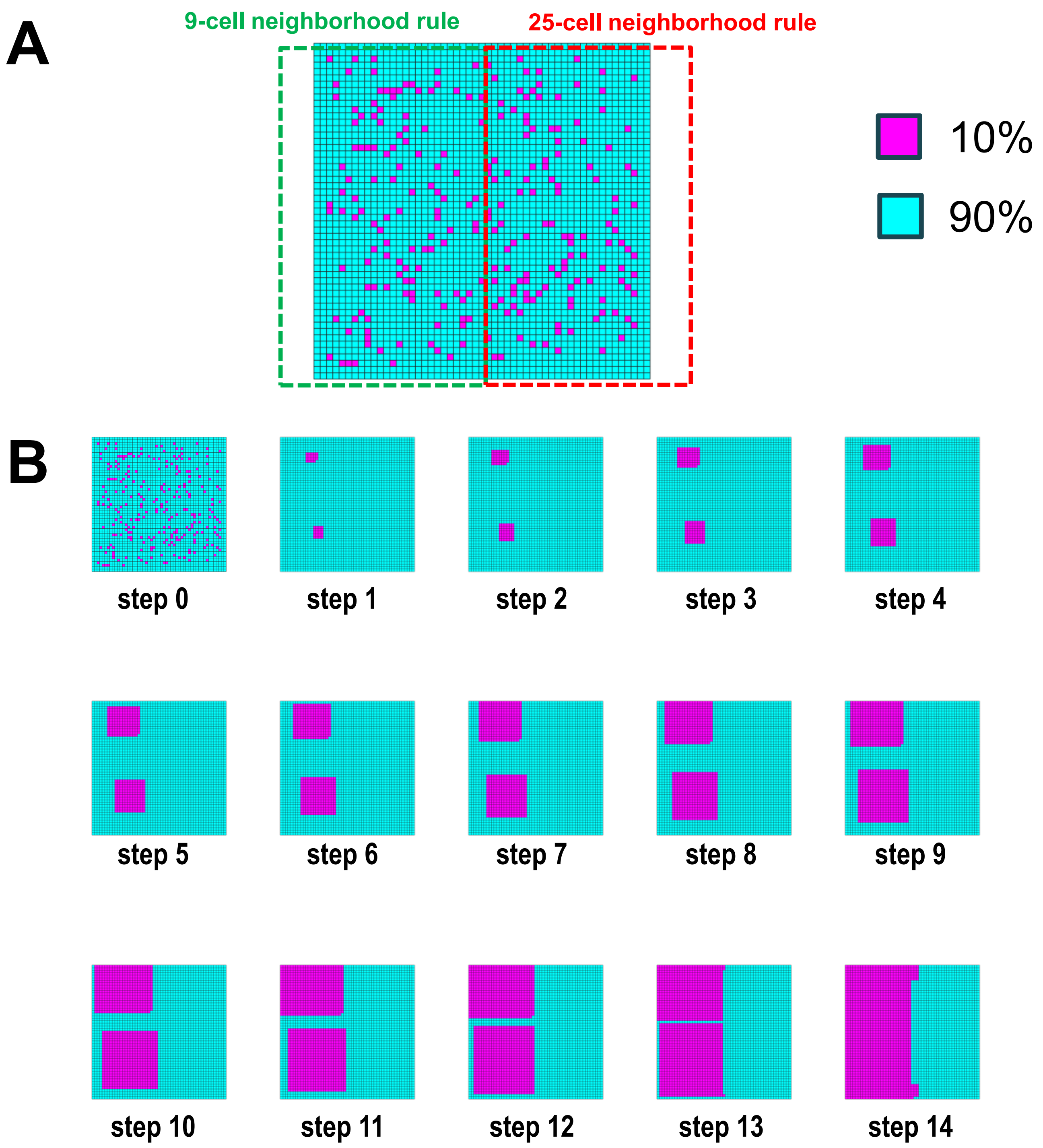


**Figure S6. Simulation on a 50 × 50 grid with a heterogeneous neighborhood rule, based on the model in Figure S6. (A)** The left half of the grid uses the 9-cell neighborhood rule, and the right half uses the 25-cell rule. This setup simulates tissue with a short space constant on the left and a long space constant on the right. **(B)** Images show time snapshots from a representative simulation starting with a random distribution of "alive" cells ("seeds"). The rule is applied iteratively, demonstrating how waves of "alive" cells can initiate and propagate across the grid.

**Figure S6**

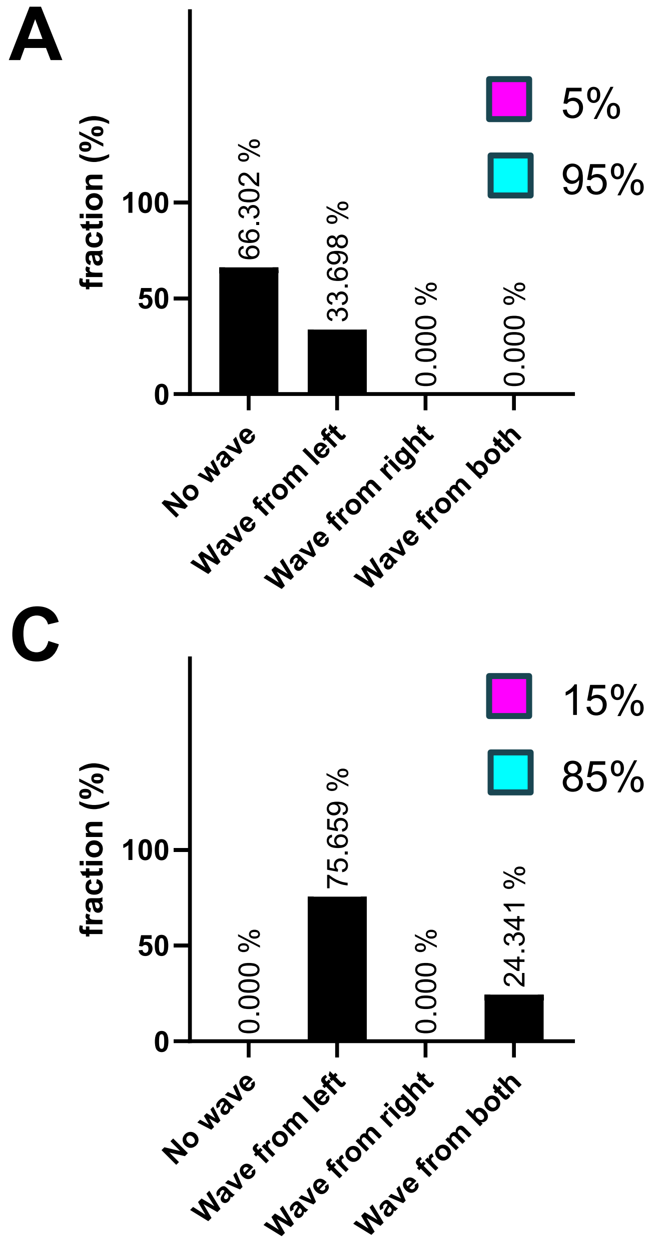


**Figure S7. Summary of simulations described in Figure S6 with different random seeds, showing how the initial density of "alive" cells affects wave initiation.** The percentage of initially "alive" cells is analogous to the density of pathological cells (DAD$^+$ cells) in tissue; a lower percentage represents healthier tissue. Simulations were run with initial "alive" cell densities ranging from 5% to 15%. **(A)** With 5% initially "alive," waves generally failed to form, and the grid tended toward a "dead" state. **(B)** With 10% initially "alive," waves were typically initiated on the left side (short space constant), because a smaller neighborhood increases the likelihood of randomly exceeding the critical mass threshold. **(C)** With 15% initially "alive," waves often initiated on the left side, but many also initiated simultaneously on the right side.

**Figure S7**

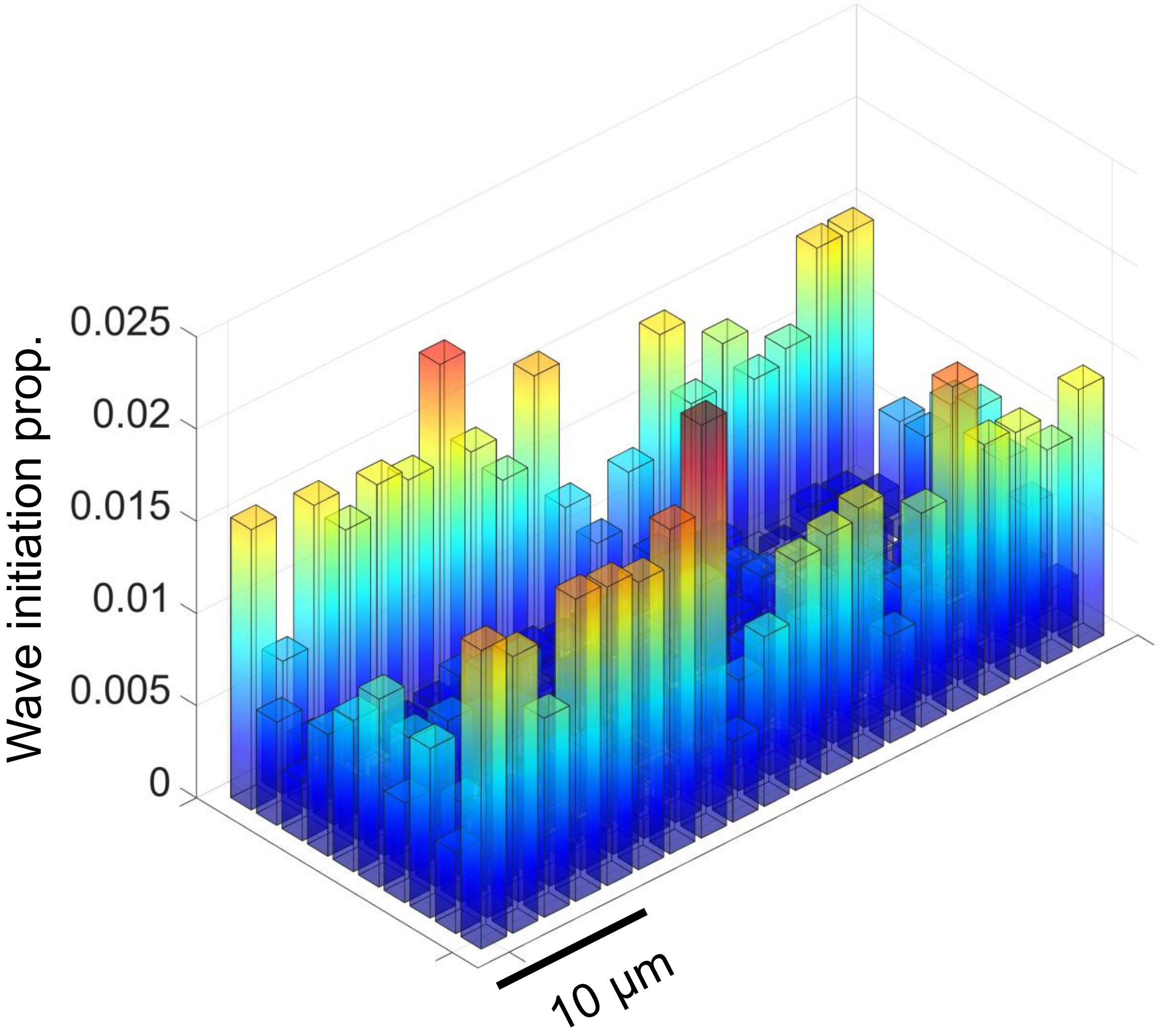


**Figure S8. $Ca^{2+}$ waves are predominantly initiated in proximity to the cell periphery.** This three-dimensional bar graph illustrates the frequency of $Ca^{2+}$ wave initiation points. Cell borders exhibit significantly higher frequencies (74% of waves originate within the outer two layers) compared to the central regions of the cell (26%), attributable to the limited sink effect near the cell periphery.

**Figure S8**
